# Genome editing with the HDR-enhancing DNA-PKcs inhibitor AZD7648 causes large-scale genomic alterations

**DOI:** 10.1038/s41587-024-02488-6

**Published:** 2024-11-27

**Authors:** Grégoire Cullot, Eric J. Aird, Moritz F. Schlapansky, Charles D. Yeh, Lilly van de Venn, Iryna Vykhlyantseva, Susanne Kreutzer, Dominic Mailänder, Bohdan Lewków, Julia Klermund, Christian Montellese, Martina Biserni, Florian Aeschimann, Cédric Vonarburg, Helmuth Gehart, Toni Cathomen, Jacob E. Corn

**Affiliations:** 1 Department of Biology, Institute of Molecular Health Sciences, ETH Zurich, Zurich, Switzerland; 2 Institute for Transfusion Medicine and Gene Therapy, Medical Center – University of Freiburg, Freiburg, Germany; 3 Center for Chronic Immunodeficiency, Faculty of Medicine, University of Freiburg, Freiburg, Germany; 4 CSL Behring Research, Bern, Switzerland; 5 Swiss Institute for Translational Medicine sitem-insel, Bern, Switzerland

**Keywords:** Homologous recombination, Non-homologous-end joining, Double-strand DNA breaks, Genetic engineering, Targeted gene repair

## Abstract

The DNA-PKcs inhibitor AZD7648 enhances CRISPR–Cas9-directed homology-directed repair efficiencies, with potential for clinical utility, but its possible on-target consequences are unknown. We found that genome editing with AZD7648 causes frequent kilobase-scale and megabase-scale deletions, chromosome arm loss and translocations. These large-scale chromosomal alterations evade detection through typical genome editing assays, prompting caution in deploying AZD7648 and reinforcing the need to investigate multiple types of potential editing outcomes.

## Abstract (web summary)

A compound that enhances homology-directed repair in CRISPR editing leads to genome instability.

## Main

Double-strand breaks (DSB) introduced using genome editing technologies can be repaired through non-homologous end joining (NHEJ), which directly ligates broken DNA ends and results in small insertions and deletions (indels)^1,2^. Alternatively, end resection enables activation of homology-based repair pathways. Microhomology-mediated end joining (MMEJ) repairs overlaps of short homologous sequences near the DSB and results in small deletions of typically less than 20 base pairs (bp). Homology-directed repair (HDR) copies information from a DNA template into a targeted locus. Desired sequences can be introduced via HDR by providing an exogenous template, with alterations ranging from single-nucleotide mutations to entire transgenes. This ability to precisely introduce DNA sequences through HDR holds promise for biomedical research and therapy.

The first CRISPR–Cas medicines leverage error-prone NHEJ and MMEJ disruption of target genes rather than HDR^2,3^. This is in part due to the low efficiency of HDR in human cells relative to the competing NHEJ and MMEJ pathways. Extensive work has been conducted to increase HDR rates through modulation of the cell cycle, tethering of the DNA repair template, repeated targeting of the genomic locus, activation of HDR-favoring pathways or inhibition of NHEJ to redirect toward HDR^4–11^.

DNA-PKcs plays a crucial role in both promoting NHEJ and repressing HDR, and inhibiting DNA-PKcs increases HDR across multiple loci and cell types^12–16^. A highly potent and selective DNA-PKcs inhibitor (AZD7648) has garnered a great deal of recent attention due to its apparent ability to significantly improve HDR efficiencies in both transformed cell lines and primary human cells^17–19^. AZD7648 has the potential to promote clinical translation of HDR-based therapies by enabling therapeutic levels of gene correction, but potential unintended consequences of its use during genome editing have not been well explored.

In this study, we investigated the effects of AZD7648 on on-target editing outcomes. We found that using AZD7648 during genome editing increased apparent HDR rates as determined by short-read sequencing in multiple cell types and across multiple loci. However, this increase in HDR was accompanied by Cas9-induced on-target genomic instability that converted small-scale NHEJ outcomes into larger genetic changes undetectable by short-read sequencing. Using long-read sequencing, droplet digital polymerase chain reaction (ddPCR)-based copy number quantification, single-cell RNA sequencing (scRNA-seq) and unbiased translocation detection, we found that AZD7648 greatly increases the occurrence of kilobase-scale deletions, chromosome arm loss and translocations in multiple cell backgrounds. Our results urge caution when deploying AZD7648 during genome editing and reinforce the need to investigate genetic outcomes beyond those accessible to short-read target amplicon next-generation sequencing (NGS).

**Fig. 1:**
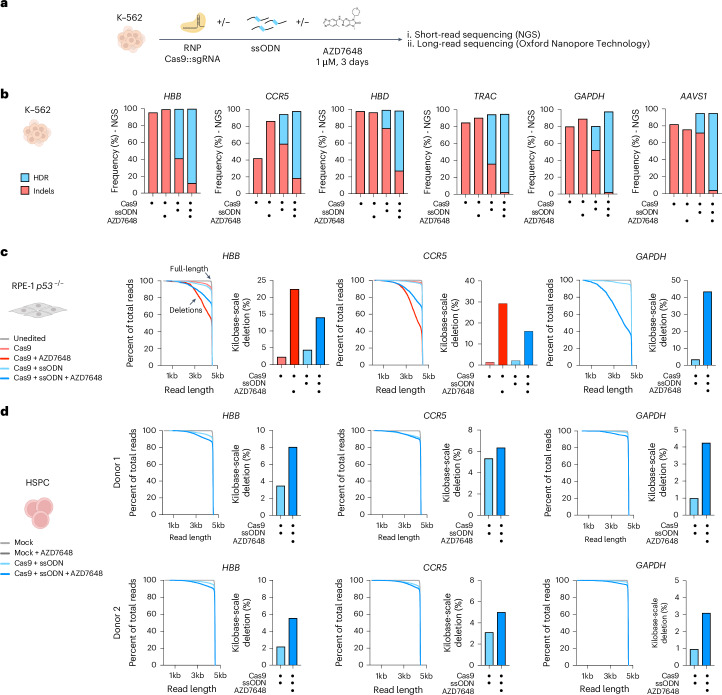
AZD7648-induced HDR gain is skewed by an increased frequency of kilobase-scale deletions. **a**, Schematic of editing workflow. K-562 cells were electroporated with a Cas9–sgRNA RNP with co-delivery of an ssODN HDR donor. After electroporation, edited cells were treated with 1 μM AZD7648 for 3 d and analyzed by short-read (Illumina) and long-read (ONT) sequencing. **b**, Frequency of HDR and indels detected by short-read sequencing in edited K-562 cells. **c**,**d**, Quantification by long-read sequencing of the fraction of total reads with a length greater than a defined size and quantified frequency of deletions larger than 1 kb in edited RPE-1 p53^−/−^ (**c**) and HSPCs (**d**). Data are from *n* = 1 biological replicate and *n* = 2 replicates with two distinct donors for the HSPC experiments.

**Extended Data Fig. 1:**
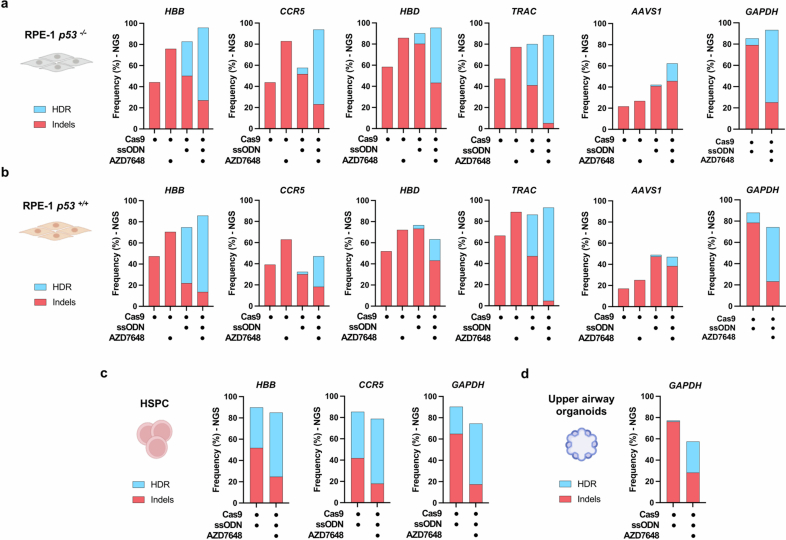
AZD7648-induced DNA-PKcs inhibition increases detected HDR in RPE-1 and primary cells. (**a-d**) Frequency of HDR and indels detected by short-read sequencing in edited RPE-1 p53^−/−^ **(a**), RPE-1 p53^+/+^ (**b**), HSPC (**c**), upper airway organoid cells (**d**). Genomic DNA was collected 3 d post-electroporation. Cells were electroporated with Cas9 in complex with a single guide RNA (sgRNA) as an RNP, with co-delivery of a single-stranded oligodeoxynucleotide (ssODN) HDR donors. Edited cells were treated with 1 μM AZD7648 for 3 d immediately following electroporation. Genomic DNA was collected 3 d post-electroporation. Data are from *n* = 1 biological replicate and one donor for HSPC experiments.

**Extended Data Fig. 2:**
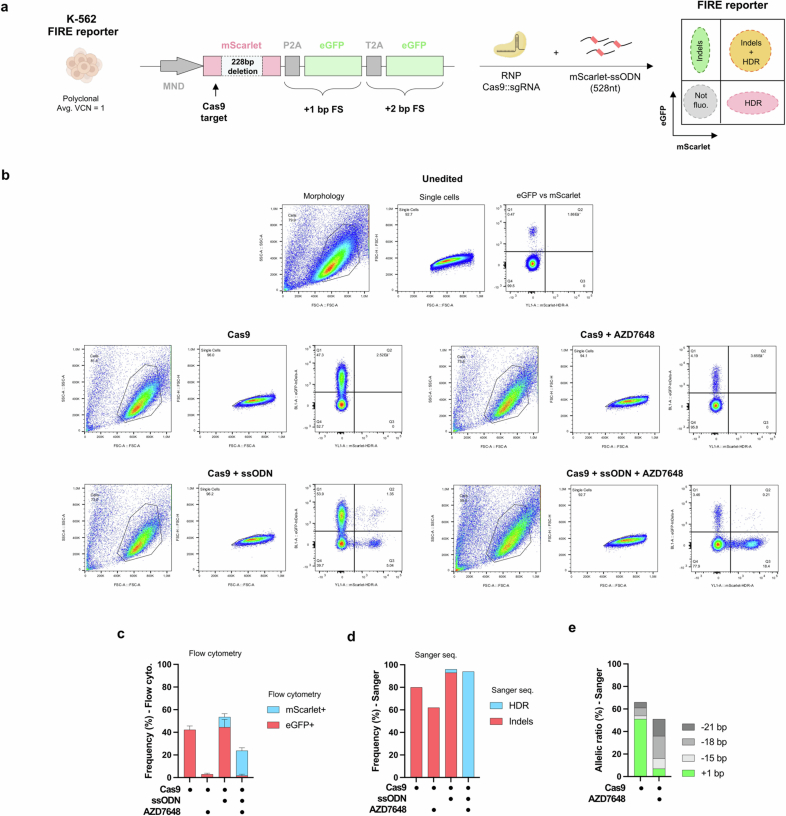
The FIRE reporter enables HDR and frame-shift indels quantification by flow cytometry. (**a**) Schematic of the FIRE reporter construct. The FIRE reporter system consists of an in-frame disrupted mScarlet sequence (with a 228 nt deletion), followed by two out-of-frame eGFP cassettes (+1 bp and +2 bp). K-562 cells with the FIRE reporter were electroporated to induce a DSB in the disrupted mScarlet sequence. The HDR template is a long 528 nt ssODN containing the missing 228 nt of the mScarlet sequence flanked by two 150 nt-homology arms. HDR-mediated repair using the corrective ssODN is expected to lead to the mScarlet expression, while generation of indels causing frameshifts should trigger eGFP expression. Unmodified target sites, in-frame small indels, reporter silencing, and larger deletions that lead to loss of the reporter expression are expected to result in non-fluorescent cells. (**b**, **c**) Flow cytometry data of edited K-562 FIRE reporter cells (**b**) and quantification of HDR (mScarlet+) and indels (eGFP+) event frequencies (**c**) detected by flow cytometry (n = 4 biological replicates). (**d**) Quantification of HDR and indel reads detected by Sanger sequencing and Inference of CRISPR Edits (ICE) in edited K-562 cells at the FIRE reporter (n = 1 biological replicate). (**e**) Indel profile in Cas9-edited K-562 FIRE reporter cells assessed by ICE analysis. Analyses were performed 3 days post-electroporation for both flow cytometry and Sanger sequencing assays.

We tested the efficacy of AZD7648 in enhancing HDR at multiple endogenous loci and found that it increased the apparent fraction of HDR alleles detected by Illumina short-read NGS (Fig. 1a,b, Extended Data Fig. 1 and Supplementary Table 1). For several loci, we found that AZD7648 treatment led to the detection of an almost pure population of HDR reads, with few remaining indels and wild-type reads. We further investigated this by developing a single-copy integrated fluorescent insertional repair and end-joining (FIRE) traffic light reporter, which tracks both out-of-frame indels and HDR outcomes through gain of fluorescence (Extended Data Fig. 2a). Using this system, we found that editing without AZD7648 led to detectable indel and HDR fluorescence events (Extended Data Fig. 2b,c). Editing with AZD7648 almost completely abolished indel events and yielded 22.0 ± 2.2% HDR events, as well as 76.0 ± 1.3% of events with no detectable fluorescence. However, analysis of the same edited cells by Sanger sequencing of the FIRE locus reported approximately 93% HDR alleles, no indels and only 7% wild-type reads (Extended Data Fig. 2d). When editing without a single-stranded oligodeoxynucleotide (ssODN), AZD7648 treatment led to an increased deletion size in the remaining indel alleles (Extended Data Fig. 2e). The discrepancy in the apparent rates of each editing outcome by phenotypic flow cytometry and Sanger sequencing suggests that AZD7648 treatment might cause allelic dropout due to the creation of larger deletions that evade short-range PCR amplification.

**Extended Data Fig. 3:**
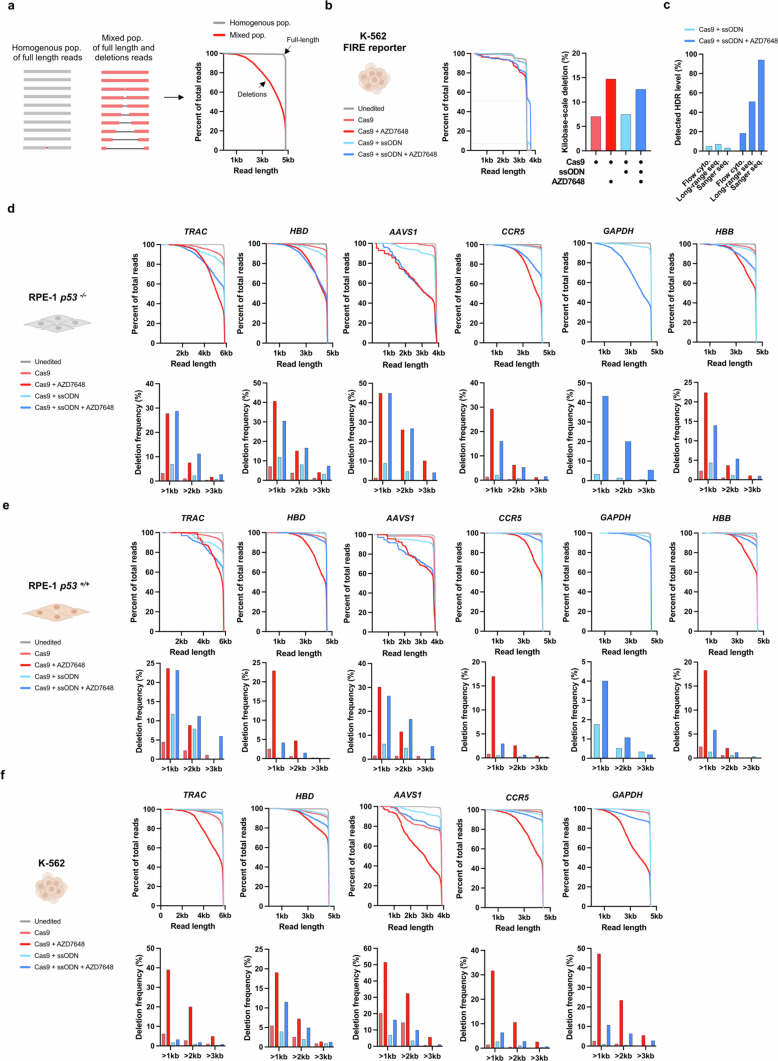
AZD7648 increases frequency of kilobase-scale deletions. (**a**) Schematic of long-read deletion quantification. Reads obtained from ONT sequencing were initially quality filtered and aligned to the target site (see Methods). The fraction of total reads of a greater length than a defined size was calculated. A population of reads with deletions leads to a leftward shift of the curve. **(b**) AZD7648 induces kilobase-scale deletions in K-562 cells at the FIRE reporter. Raw data is shown in the left panel, and the quantified frequency of deletions larger than 1 kb is shown in the right panel. **(c**) Comparison of detected HDR events using three different readouts in K-562 cells with FIRE reporter edited with Cas9 + ssODN only (light blue) or treated with AZD7648 (dark blue). **(d**–**f**) Quantification of the fraction of total reads with a length greater than a defined size (top panel) and frequency of deletions larger than 1 kb, 2 kb or 3 kb (bottom panel) in edited RPE-1 p53^−/−^ (**d**) RPE-1 p53^+/+^ (**e**) and K-562 (**f**). In all conditions, genomic DNA was collected 3 d post-electroporation. Data are from *n* = 1 biological replicate.

To discover editing outcomes that were eliminated from short-read sequencing, we re-analyzed samples from the same FIRE reporter experiment using long-range PCR amplification of a 3.5-kb window around the target site, followed by Oxford Nanopore Technologies (ONT) long-read sequencing (Extended Data Fig. 3a). After AZD7648 treatment, we found that the frequency of kilobase-scale deletions doubled from 7.5% to 14.7% (Extended Data Fig. 3b). Long-read HDR rates (assessed by the characteristic 228-bp insertion of the HDR template) were 7% without AZD7648 and approximately 50% with AZD7648. HDR levels detected using both long-read sequencing and the phenotypic FIRE assay indicate that AZD7648 does increase bona fide HDR (Extended Data Fig. 3c). However, the apparent increases in HDR levels detected by sequencing are likely inflated due to accompanying kilobase-scale deletions and larger rearrangements that interfere with amplification of the target site.

To test whether AZD7648 also increases kilobase-scale deletions at endogenous sites, we performed long-read sequencing (PCR amplicons between 3.8 kb and 5.9 kb) at the targets previously analyzed by short-read sequencing. In RPE-1 p53-null cells, we consistently observed low-level kilobase-scale deletions after gene editing without AZD7648 (Fig. 1c and Extended Data Fig. 3d). Adding AZD7648 markedly increased the frequency of kilobase-scale deletions, between 2.0-fold and 35.7-fold depending on the locus, reaching 43.3% of reads at *GAPDH*. The size of the large deletions was also increased at most loci (Extended Data Fig. 3d). We found similar results in p53-proficient RPE-1 cells and K-562 cells (Extended Data Fig. 3e,f). The frequency of large deletions was generally lower in p53^+^ cells but still greater than 20% at several loci (Extended Data Fig. 3e). We, therefore, tested whether primary cells exhibit large deletions when edited with AZD7648. Indeed, editing human CD34^+^ hematopoietic stem and progenitor cells (HSPCs) from two distinct healthy donors revealed that AZD7648 increased the frequency of large deletions by 1.2-fold to 4.3-fold at three target loci (Fig. 1d).

**Fig. 2:**
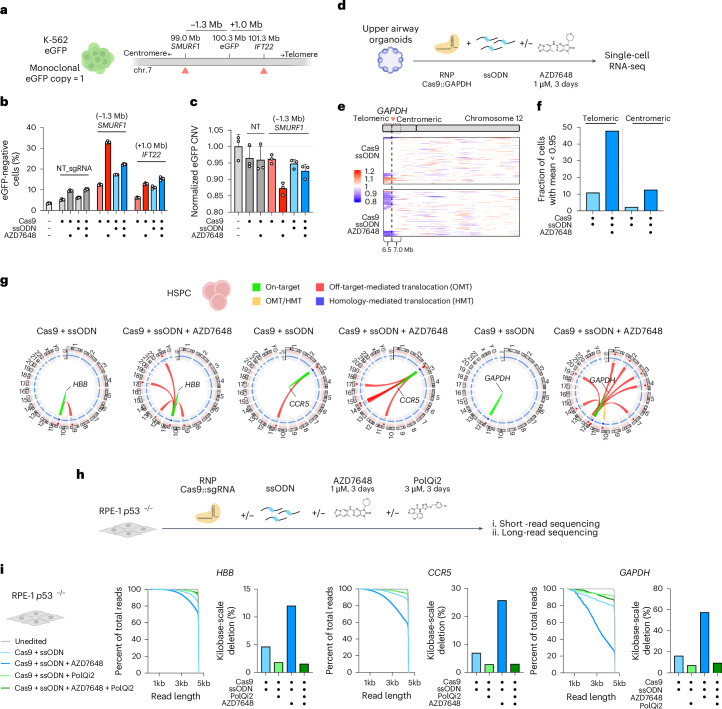
AZD7648 increases the frequency of megabase-scale deletions, chromosome arm loss and translocations. **a**, Schematic of chromosome 7 around the eGFP insertion site in clonal K-562 cells, showing the *eGFP* location, −1.3-Mb editing site (located in *SMURF1*) and the +1-Mb editing site (located in *IFT22*). Cas9 RNP target sites are marked by red triangles. **b**, Quantification of the eGFP^−^ cell fraction detected by flow cytometry. **c**, K-562 eGFP cells edited at −1.3 Mb or with a non-targeting (NT) guide were analyzed by ddPCR to quantify copy numbers of the eGFP cassette in total edited cells. Copy number variation (CNV) is represented by the ratio of the eGFP copy number divided by the copy number detected at a control site on a different chromosome (chr3:46,270,956–46,271,086) and normalized to the unedited condition. In all conditions, gDNA was collected 6 d after electroporation. **d**, Schematic of the workflow for editing upper airway organoids. **e**, Heatmap of residual gene expression of *GAPDH* UTR-edited upper airway organoids. **f**, Frequency of upper airway organoid cells exhibiting gene expression loss in the 6.5-Mb telomeric or 7.0-Mb centromeric segment. Cells with average residual gene expression lower than 0.95 were considered as cells with gene expression loss^23^. **g**, Circos plots of CAST-seq-detected chromosomal translocations in edited HSPCs^25^. **h**, Schematic of the workflow for editing RPE-1^−/−^ cells with AZD7648 and PolQi2 in combination. **i**, Quantification by long-read sequencing of the fraction of total reads with a length greater than a defined size and quantified frequency of deletions larger than 1 kb in edited RPE-1 p53^−/−^. Data are from *n* = 1 biological replicate (with three technical replicates for **c**), except for **b** with *n* = 4 biological replicates. **b**,**c**, Results are presented as mean ± s.d.

Genome editing can induce megabase-scale chromosomal aberrations in both immortalized and primary cells, even in the absence of AZD7648 (refs. ^20–22^). To investigate whether AZD7648 treatment influenced the frequency of very large genomic effects that would remain undetected by even long-read sequencing, we used a clonal K-562 cell line with a single integrated copy of eGFP (Fig. 2a). Editing either −1.3 Mb toward the centromere (*SMURF1*) or +1.0 Mb toward the telomere (*IFT22*) relative to the eGFP insertion site, we assessed local and chromosome-scale outcomes using a combination of short-read sequencing at the target sites and eGFP fluorescence.

**Extended Data Fig. 4:**
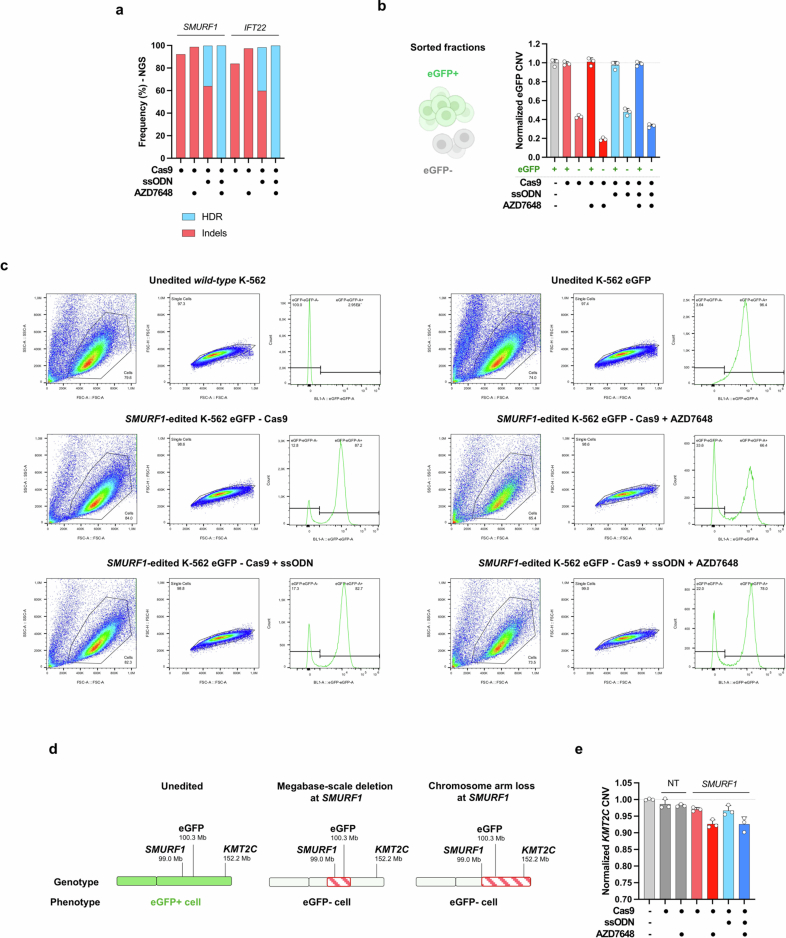
AZD7648 increases frequency of megabase-scale deletions and chromosome arm loss. (**a**) Frequency of HDR and indels detected by short-read sequencing in K-562 eGFP cells edited at −1.3 Mb or +1 Mb relative to the reporter. (**b**, **c**) K-562 eGFP cells edited at −1.3 Mb were analyzed by droplet digital PCR to quantify copy numbers of the *eGFP* cassette in fractions sorted for eGFP+ and eGFP- cells with gating strategy shown in (**c**). (**d**) Schematic representation of chromosome 7 of −1.3 Mb-edited K-562 eGFP clonal cell line, illustrating potential outcomes editing at −1.3 Mb (**e**) K-562 eGFP cells edited at −1.3 Mb or with a non-targeting guide (NT) were analyzed by droplet digital PCR to quantify copy numbers of *KMT2C*. Edited cells were analyzed 6 d post-electroporation. Copy number variation (CNV) is represented by the ratio of *eGFP* (**b**) or *KMT2C* (**e**) copy numbers divided by the copy number detected at chr3:46,270,956-46,271,086, normalized to the unedited condition. Data are from *n* = 1 biological replicate with three technical replicates in (**b**) and (**e**). (**b**, **e**) Results are presented as mean ± standard deviation.

We found that AZD7648 treatment resulted in the detection of solely HDR reads at the Cas9-targeted site by short-read sequencing when editing at the −1.3-Mb site or the +1-Mb site (Extended Data Fig. 4a). By flow cytometry, we found that AZD7648 led to up to 33 ± 0.1% of cells when editing −1.3 Mb from the *eGFP* site, with smaller changes when editing at +1.0 Mb (Fig. 2b). Using ddPCR, we confirmed that editing with AZD7648 at the −1.3-Mb site resulted in eGFP bulk averaged copy number fractional loss of −0.14 ± 0.018, with sorted eGFP^−^ cells completely losing their genomic copy of *eGFP* (Fig. 2c and Extended Data Fig. 4b,c). The observation that editing on the centromeric side of the *eGFP* led to reporter loss, whereas editing on the telomeric side did not, suggests that the editing may have caused chromosome arm loss. To determine if deletions causing *eGFP* loss could extend further, potentially resulting in the loss of the entire chromosome arm, we performed ddPCR copy number quantification at *KMT2C* (+52 Mb) after editing at the −1.3-Mb site (Extended Data Fig. 4d). Strikingly, in the presence of AZD7648, we observed +52-Mb copy number fractional loss of up to −0.074 ± 0.012 (Extended Data Fig. 4e).

**Extended Data Fig. 5:**
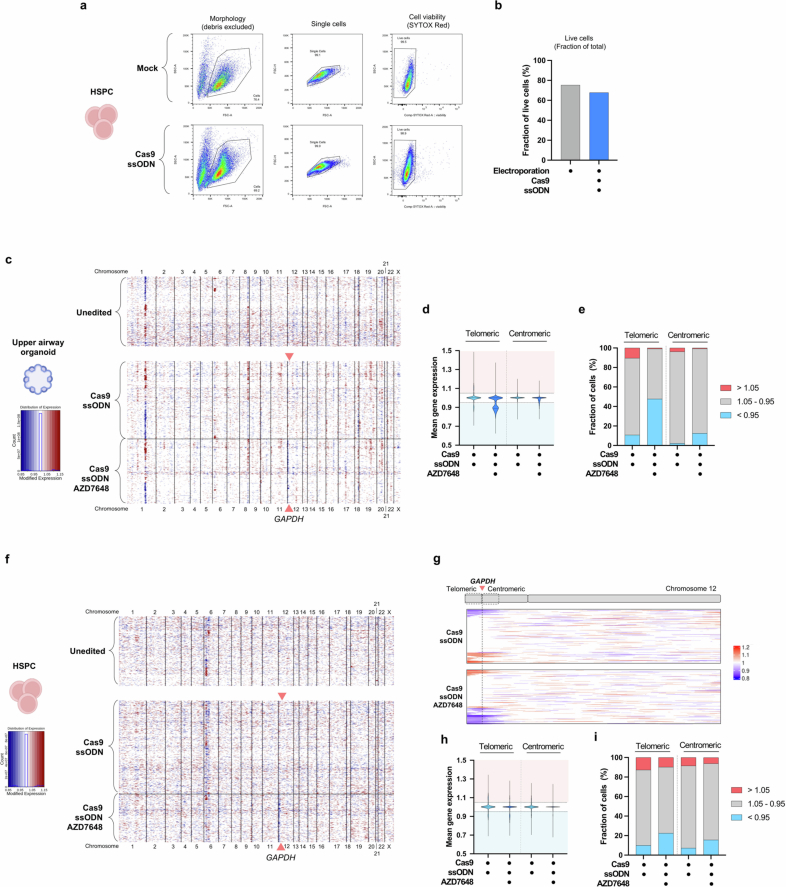
AZD7648 increases the frequency of gene expression loss on the targeted chromosome arm in primary cells and organoids. (**a**, **b**) Flow cytometry data (**a**) and quantification (**b**) of cell viability of *GAPDH* UTR-edited (blue) or mock-electroporated (grey) HSPCs. The fraction of live cells is calculated as the ratio of SYTOX Red-negative cells to the total number of events analyzed. (**c**) Heatmap of residual gene expression of UTR-edited upper airway organoids across the entire genome. (**d**, **e**) Mean normalized gene expression of edited upper airway organoid cells (**d**) and frequency of upper airway organoid cells with gene expression gain (average residual gene expression > 1.05 – in red), unmodified gene expression (from 1.05 to 0.95), or gene expression loss (< 0.95 – in blue) (**e**) in the telomeric or centromeric segment of the *GAPDH* UTR target site. (**f**, **g**) Heatmap showing the residual gene expression of UTR-edited HSPCs across the entire genome (**f**) and specifically on chromosome 12 (**g**). (**h**, **i**) Mean normalized expression of transcripts (**h**) and frequency of HSPCs with gene expression gain (average residual gene expression > 1.05 – in red), unmodified gene expression (from 1.05 to 0.95), or gene expression loss (< 0.95 – in blue) (**i**) in the telomeric or centromeric segment of the *GAPDH* target site for edited HSPCs. The number of analyzed cells per condition in (**c**) and (**f**) ranged from 9,296 to 14,871. The red triangles indicate the Cas9 RNP target sites. Telomeric and centromeric segments were defined as the chromosome start to the *GAPDH* target site (chr12:6,535,922) and the 7.0 Mb region immediately downstream of the cut site. Data are from *n* = 1 biological replicate.

To gain deeper insights into the effect of AZD7648 treatment on large-scale genomic alterations in primary cells, we edited upper airway organoids and human HSPCs in the 3′ untranslated region (UTR) of *GAPDH*, confirming that the edit did not greatly affect overall cell health (Extended Data Fig. 5a,b). We then conducted scRNA-seq on the edited cells (Fig. 2d–f and Extended Data Fig. 5c–i). In this experiment, loss of coherent blocks of RNA expression around a Cas9 target site is indicative of copy number alteration^23^.

Analyzing approximately 40,000 individual cells, we found that editing with AZD7648 markedly increased gene expression loss of a 6.5-Mb telomeric segment in both upper airway organoids (Fig. 2e) and HSPCs (Extended Data Fig. 5g). Up to 47.8% of upper airway organoid cells and 22.5% of HSPCs exhibited gene expression loss in a pattern consistent with loss of the chromosome arm spanning to the telomere and encompassing 24 genes (Fig. 2f and Extended Data Fig. 5h–i).

**Extended Data Fig. 6:**
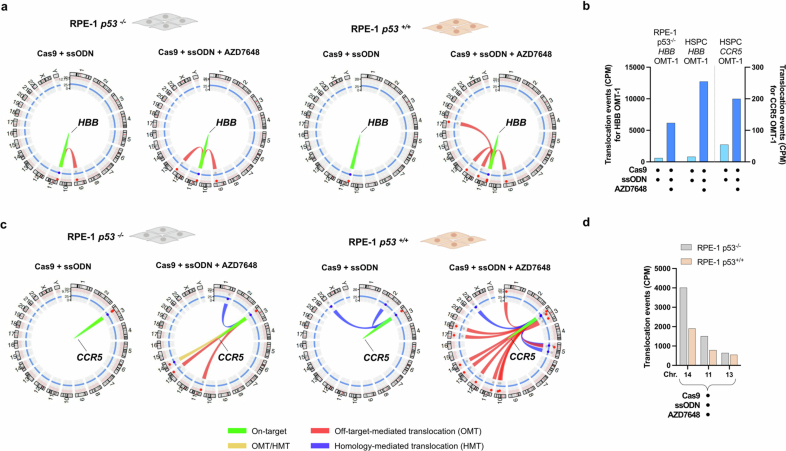
AZD7648 increases the frequency of translocations. (**a**) Circos plots of detected chromosomal translocations in RPE-1 p53^−/−^ (left panel) and RPE-1 p53^+/+^ (right panel) edited at *HBB*. On-target aberrations are shown in green, off-target mediated translocations (OMTs) in red, homology-mediated translocations (HMTs) in blue, and mixed types in yellow^25^. (**b**) Quantification of translocation events. Shown is the number of reads (counts per million, CPM) for *HBB* OMT-1 (chr.9) in RPE-1 p53^−/−^ and HSPCs edited at *HBB* with or without AZD7648 and for *CCR5* OMT-1 (chr. 11) in HSPCs edited at *HBB* with or without AZD7648 normalized by the total number of reads. (**c**) Circos plots of detected chromosomal translocations in RPE-1 p53^−/−^ (left panel) and RPE-1 p53^+/+^ (right panel) edited at *CCR5*. (**d**) Quantification of translocation events. Indicated is the number of reads (counts per million, CPM) for OMT-1 (chr.14), OMT-2 (chr.11) and OMT-3 (chr.13) in *CCR5*-edited RPE-1 p53^−/−^ or RPE-1 p53^+/+^ with AZD7648. Data are from *n* = 1 biological replicate with *n* = 2 technical replicates, except for RPE-1 p53^+/+^ edited at *CCR5* (*n* = 1).

Targeting two loci simultaneously in the presence of AZD7648 was previously shown to favor translocations in HEK293T and K-562 cells^17,24^. We, therefore, asked whether using AZD7648 might increase the frequency of translocations when targeting a single locus with single guide RNA (sgRNA). We individually edited CD34^+^ HSPCs using three distinct guide RNAs and used CAST-seq for unbiased detection of translocations^25,26^. Editing with AZD7648 markedly increased the number of detected translocations (Fig. 2g and Supplementary Table 2). We observed similar results in p53-null and p53-proficient RPE-1 cells, where AZD7648 increased both the number of translocation sites as well as the frequency of translocation at each site (Extended Data Fig. 6a–d), especially in p53-null cells.

Kilobase-scale deletions can exhibit microhomologies, suggesting repair via the MMEJ pathway^27^. DNA polymerase theta (Polθ) plays a crucial role in MMEJ and can be effectively inhibited with PolQi2, a potent inhibitor of its helicase activity^17,28^. We tested whether editing with PolQi2 can prevent the large deletions and rearrangements caused by AZD7648 treatment (Fig. 2h).

**Extended Data Fig. 7:**
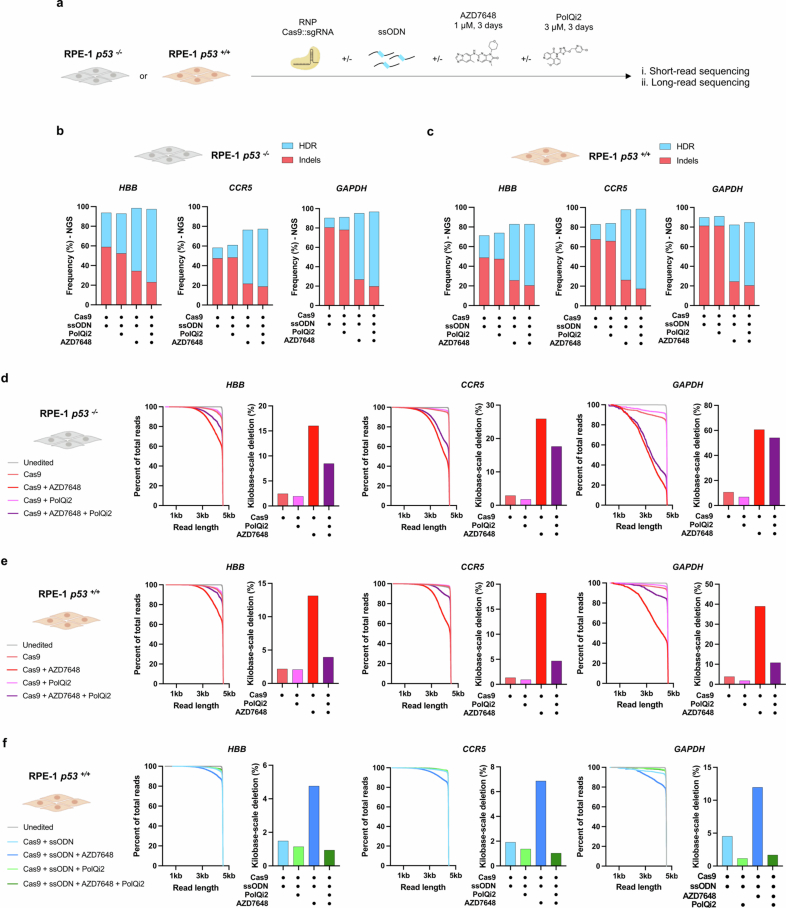
Addition of PolQi2 preserves high HDR and limits occurrence of AZD7648-induced kilobase-scale deletions. (**a**) Schematic of the editing workflow. RPE-1 p53^−/−^ and RPE-1 p53^+/+^ cells were electroporated with a Cas9 RNP, with co-delivery of a ssODN HDR donor. Edited cells were treated with 1 μM AZD7648 and/or 3 μM PolQi2 for 3 d immediately following electroporation. (**b**, **c**) Frequency of HDR and indels detected by short-read sequencing in RPE-1 p53^−/−^ (**b**) and RPE-1 p53^+/+^ cells (**c**) edited at *HBB*, *CCR5* and *GAPDH*. Genomic DNA was collected 3 d post-electroporation. (**d**–**f**) Quantification by long-read sequencing of the fraction of total reads with a length greater than a defined size and quantified frequency of deletions larger than 1 kb in RPE-1 p53^−/−^ edited without the ssODN HDR donor (**d**) and RPE-1 p53^+/+^ edited without (**e**) or with the ssODN HDR donor (**f**). Data are from *n* = 1 biological replicate.

**Extended Data Fig. 8:**
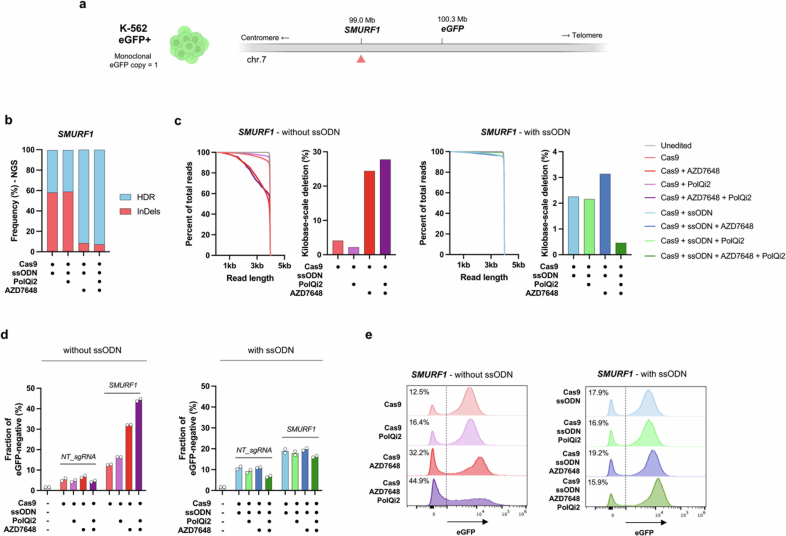
AZD7648 and PolQi2 in combination did not reduce chromosome arm loss when co-delivered with an ssODN HDR template. (**a**) Schematic of the region of chromosome 7 surrounding the eGFP insertion site in a clonal K-562 cell line. The position of the single-copy eGFP insertion site and of the *SMURF1* (−1.3 Mb) locus are indicated. The red triangle represents the Cas9 RNP target site. (**b**) Frequency of HDR and indels detected by short-read sequencing in K-562 eGFP cells edited at *SMURF1*. Genomic DNA was collected 6 d post-electroporation. (**c**) Quantification by long-read sequencing of the fraction of total reads with a length greater than a defined size and quantified frequency of deletions larger than 1 kb in K-562 eGFP edited at *SMURF1* in the absence of ssODN delivery (left panel) and in its presence (right panel). (**d**, **e**) Quantification (**d**) and illustrative flow cytometry data (**e**) of the eGFP-negative cell fraction. Edited cells were analyzed 6 d post-electroporation. Data are from *n* = 1 biological replicate, except for (**d**) with *n* = 2 biological replicates.

We found that editing with combined AZD7648 and PolQi2 maintained elevated HDR levels (Extended Data Fig. 7a–c) while reducing AZD7648-induced kilobase-scale deletions across multiple loci and cell types (Fig. 2i and Extended Data Fig. 7d–f). When editing the K-562 eGFP reporter cells at the −1.3-Mb site (Extended Data Fig. 8a), we also found that PolQi2 reduced AZD7648-induced kilobase-scale deletions (Extended Data Fig. 8b,c). However, in this same experiment, PolQi2 did little to ameliorate the megabase-scale deletions that extend on the telomeric side to remove the eGFP reporter (Extended Data Fig. 8d,e).

Overall, our data indicate that editing with an sgRNA in combination with the HDR-enhancing DNA-PKCs inhibitor AZD7648 induces kilobase-scale and megabase-scale deletions and translocations in multiple human cell types. These on-target effects were previously associated with less selective DNA-PKcs inhibitors, suggesting that DNA-PKcs inhibition as a general class may be prone to such outcomes in human cells^12–15^. It remains to be seen if triple treatment with a Cas9–sgRNA complex to edit, AZD7648 to increase HDR and PolQi2 to ameliorate induced kilobase-scale deletions will be a viable path forward for genome editing applications beyond fundamental research, especially because PolQi2 so far does not seem to improve megabase-scale unintended consequences of AZD7648.

Genome editing can be associated with on-target outcomes that extend beyond the immediate vicinity of the target site. Despite constituting a non-negligible fraction of repair, these larger events can be difficult to detect and are invisible to short-read sequencing, which is commonly employed to assess genome editing outcomes^20,22,29^. These overlooked consequences can extend beyond DSB-introducing genome editors, as base editing in HSPCs has recently been reported to be associated with translocations^30,31^. Further investigation may be warranted when inhibiting other DNA repair pathways, for example, when inhibiting mismatch repair to boost prime editing efficiency^32,33^.

## Methods

### Cell culture

K-562 cells were cultured in RPMI medium (Gibco, 11875093) supplemented with 10% FBS and 100 µg ml^−1^ penicillin–streptomycin (Gibco, 15140122).

hTERT-immortalized retinal pigmental epithelium (hTERT RPE-1) cell lines were cultured in DMEM, supplemented with 10% FBS and 100 µg ml^−1^ penicillin–streptomycin. The RPE-1 cell line invalidated for TP53 (RPE-1 p53^−/−^) was a gift from Stephen Jackson^34^.

For scRNA-seq experiments, human G-CSF-mobilized CD34^+^ HSPCs from adult healthy donors were purchased from the Fred Hutchinson Cancer Center and cultured in StemSpan SFEM II media (STEMCELL Technologies, 09655) supplemented with StemSpan CC110 (1×) (STEMCELL Technologies, 02697) and 100 µg ml^−1^ penicillin–streptomycin.

For editing experiments, mobilized human peripheral blood CD34^+^ HSPCs (STEMCELL Technologies) were cultured in StemSpan SFEM II medium supplemented with 100 ng µl^−1^ each of FLT3-L, TPO and SCF (Miltenyi Biotec, 130-096-474, 130-095-745 and 130-096-491) as well as 750 nM SR1 and 35 nM UM729 (STEMCELL Technologies, 72342 and 72332).

During routine culture, human upper airway organoids were seeded in six-well plates in 15 μl of basement membrane extract (BME) droplets and polymerized for 30 min at 37 °C. After BME solidification, organoid-specific culture medium (Supplementary Table 3) was added, and the organoids were cultured at 37 °C until further use. For each passage (every 4–6 d), the organoids were dissociated mechanically, washed to remove remaining BME and seeded at half the density in fresh BME. The human nasal epithelial samples were collected from healthy volunteers, in accordance with ethical guidelines of the ETH Zürich Ethics Commission (EK-2024-N-171-A) and the Cantonal Ethics Committee Zürich (Req-2O24-OO558).

The hTERT RPE-1 p53^+/+^, hTERT RPE-1 p53^−/−^, K-562 and K-562 eGFP cell lines were short tandem repeat (STR) profiled and tested negative for mycoplasma. All cells were cultured at 37 °C and 5% CO_2_ in a humidified chamber.

### FIRE reporter generation

The polyclonal FIRE reporter cell line was generated by lentiviral transduction of K-562 cells. In brief, lentiviruses were produced by transient transfection of a lentiviral transfer plasmid containing the reporter construct as well as a G418 resistance cassette into the GPRTG lentivirus producer cell line^35,36^. The lentiviral vector used in this study for generation of the polyclonal FIRE reporter is derived from the pCL20c vector backbone^35^. Sequences of interest were generated by gene synthesis and cloned into the vector backbone by GenScript.

Lentivirus-containing supernatant was collected 2 d after transfection, filtered through a 0.22-µm filter and used for transduction of K-562 cells at different dilutions to generate polyclonal cell lines with different vector copy numbers (VCNs). Four days after transduction, cells were treated with 800 µg ml^−1^ G418 for 7 d, after which cells were cultured in the presence of 400 µg ml^−1^ G418. After 7 d of initial selection, genomic DNA (gDNA) was extracted to measure the average VCN of integrated sequences by ddPCR. Cells with an average VCN of approximately 1 were selected for subsequent experiments.

### Editing reagents

Editing reagents used are summarized in Supplementary Table 1. All experiments were conducted with in-house-produced SpCas9–NLS (40 µM) using previously published protocols, except for HSPC editing for which Alt-R S.p. Cas9 Nuclease V3 (Integrated DNA Technologies (IDT)) was used^37^. Synthetic guide RNAs were ordered from IDT (Alt-R CRISPR–Cas9 sgRNA) or Synthego (CRISPRevolution sgRNA EZ Kit) or produced by in vitro transcription (IVT) as described here: 10.17504/protocols.io.n2bvjyp5vk5w/v17 (ref. ^38^). For IVT, overlapping oligomers containing a T7 promoter, the desired protospacer and gRNA scaffold were amplified using Phusion polymerase (New England Biolabs (NEB), M0530L). The unpurified DNA product was then subjected to IVT using an NEB HiScribe T7 High Yield RNA Synthesis Kit (NEB, E2040L), incubating at 37 °C for 16 h. The next day, RNA was treated first with DNase I followed by recombinant Shrimp Alkaline Phosphatase (NEB, M0371S), purified with an miRNeasy kit (Qiagen, 217084), concentration measured by a NanoDrop 8000 spectrophotometer and frozen at −80 °C. ssODNs were ordered from IDT (Alt-R HDR Donor Oligo), GenScript (GenExact single-stranded DNA) or Microsynth. AZD7648 was purchased from Selleck Chemicals (S8843) and PolQi2 from MedChemExpress (HY-150279).

### Electroporation and DNA repair inhibition

Cells were edited by electroporation of a Cas9–sgRNA ribonucleoprotein (RNP) with co-delivery of ssODN when specified using 4D-Nucleofector X Unit (Lonza, AAF-1003X). Number of electroporated cells, amount of editing reagents, nucleofection kit and program used per cell type are indicated in Supplementary Table 4.

In brief, RNPs were initially formed by combining the Cas9 protein with sgRNA and incubating the mixture for 10 min at room temperature. Subsequently, ssODNs were added to the RNPs.

For editing of K-562 and RPE-1, cells were pelleted and washed once with PBS before being resuspended in the nucleofection solution. Electroporations were conducted in 20-μl Nucleocuvette Strips or in 100-μl single Nucleocuvettes using Lonza 4D electroporation kits (Supplementary Table 4). After electroporation, cells were allowed to recover for 10 min at room temperature before being transferred to six-well plates or Petri dishes containing media and 1 µM AZD7648 and 3 µM PolQi2 where indicated for 3 d before gDNA collection.

For editing of HSPCs, cells were thawed and cultured at a concentration of 0.25 × 10^6^ cells per milliliter for 48 h before electroporation. RNP and cells were prepared for electroporation as described above, and transfections were conducted in 20-µl strips (2 × 10^5^ cells per well) in triplicates. Immediately after electroporation, 80 µl of culture medium was added to each well, and cells were allowed to recover for 5 min at 37 °C before being transferred to 48-well plates containing medium (final concentration of 1 × 10^6^ cells per milliliter) and 1 µM AZD7648 where indicated. Twenty-four hours after electroporation, cells were diluted by the addition of 600 µl of culture medium. Three days after electroporation, replicates were pooled before genomic DNA extraction.

Human HSPCs for scRNA-seq were electroporated 1 d after thawing in 100-μl single Nucleocuvette (1 × 10^6^ cells per cuvette). Edited cells were collected 48 h after electroporation for scRNA-seq library preparation.

For editing experiments, upper airway organoids were extracted from BME by pre-treatment with 2 U ml^−1^ Dispase II (Gibco, 17105041) for 30 min at 37 °C, followed by mechanical disruption and digestion with TrypLE (Gibco, 12604021) for 30 min at 37 °C. After digestion, cells were washed and strained through a 70-µm strainer and counted. In total, 1 × 10^6^ cells were then washed with PBS and resuspended in SE nucleofection buffer containing Cas9, the guide RNA and ssODN (Supplementary Table 4). For cells to recover after the electroporation, complete warm culture medium was added to the cuvettes, and cells were incubated for 15 min at room temperature. After that, cells were transferred from the cuvettes to microcentrifuge tubes and pelleted (300*g* for 3 min). Afterwards, the cell pellet was resuspended in cold BME and plated as drops containing 25–100 × 10^5^ cells per drop. The cells were then cultured in complete culture medium containing 10 µM Y-27632 (TOCRIS, 1254) with or without AZD7648. After 3 d, cells were extracted from BME by mechanical disruption and TrypLE digestion and immediately subjected to the downstream analysis.

### Flow cytometry

Edited K-562 cells expressing the FIRE reporter were transferred 3 d after electroporation to a 96-well plate and analyzed for mScarlet and eGFP expression using an Attune NxT Flow Cytometer (Thermo Fisher Scientific). Cells were first gated on morphology (FSC-A versus SSC-A), for single cells (FSC-A versus FSC-H) and then on mScarlet and eGFP expression. The gating strategy is shown in Extended Data Fig. 2b.

Edited K-562 cells expressing eGFP were transferred 6 d after electroporation to a 96-well plate and analyzed for eGFP expression using an Attune NxT Flow Cytometer and Attune NxT Software version 3.2.1. Cells were first gated on morphology (FSC-A versus SSC-A), for single cells (FSC-A versus FSC-H) and then on eGFP expression. The gating strategy is shown in Extended Data Fig. 4c. Cell sorting of eGFP^+^ and eGFP^−^ fractions was performed using an SH800S Cell Sorter (Sony) and Cell Sorter Software version 2.1.6. Cell viability of *GAPDH*-edited HSPCs analyzed by scRNA-seq was also quantified using SYTOX Red Dead Cell Stain (1:1,000 dilution) (Invitrogen, S34859). Flow cytometry data were analyzed in FlowJo 10.8.1.

### gDNA extraction

gDNA was extracted 3 d after electroporation using a DNeasy Blood and Tissue Kit (Qiagen, 69504) following the manufacturer’s instructions or using phenol-chloroform for *GAPDH*-edited RPE-1 and human upper airway organoids. For phenol-chloroform extraction, one volume of phenol:chloroform:isoamyl alcohol (25:24:1) was added to the sample to extract DNA into the aqueous phase, and ethanol together with ammonium acetate was used to precipitate the purified DNA. For HSPCs, gDNA was extracted with a Maxwell RSC Cultured Cell DNA Kit (Promega, AS1620) according to the manufacturer’s instructions. All DNA samples were quantified by using the NanoDrop 8000 spectrophotometer or Qubit fluorometers (APP 2.02 + MCU version 0.26) (Thermo Fisher Scientific).

### Amplicon sequencing

Primer sequences used for amplicon sequencing are listed in Supplementary Table 5. Primers were designed to amplify a 150–250-bp region surrounding the cut site. gDNA was amplified (25× PCR cycles) using NEBNext High-Fidelity 2× PCR Master Mix (NEB, M0541L) and locus-specific primers including Illumina adapter sequences. Indexing PCR was then performed by amplifying (8× cycles) 10 ng of generated PCR products using NEBNext High-Fidelity 2× PCR Master Mix and primers including i7 / i5 Illumina indexes. PCR products were purified using 1.8× homemade SPRI beads after each PCR and quantified using Quant-iT dsDNA Assay Kits (Thermo Fisher Scientific, Q33120) and a VICTOR Nivo Microplate Reader with control software version 4.0.7 (PerkinElmer) or Qubit 4.0 Fluorometer (APP 2.02 + MCU version 0.26). After indexing PCR, samples were normalized and pooled before a final purification step using 0.8× SPRI beads. The samples were sequenced either with a MiSeq 2 × 150 paired-end or a NextSeq 2000 2 × 150 paired-end (Illumina) with a target average read count per amplicon of 200,000 reads. Illumina reads were demultiplexed and analyzed with CRISPResso2 (version 2.0.20b) in batch mode and with a quantification window size of 30 bp and default settings for the remaining parameters^39^. For indels and HDR quantification, reads with frequencies lower than 0.2% were excluded, and partial HDR reads were aggregated with the HDR fraction.

For *GAPDH*-edited RPE-1 samples, a 12-nucleotide substitution barcode was employed to uniquely tag HDR events. Reads were categorized into three groups. Initially, HDR and unedited reads were isolated using specific sequence anchors (‘CCCCCACCACACTGAATCTC’ and ‘AGAGGGGAGGGGCCTAGGGA’) with a fixed distance of 32 bp between them. The remaining reads were extracted and subjected to CRISPResso2 analysis in batch mode to determine indel frequency. From the selected reads, HDR reads were differentiated from unedited reads based on a Hamming distance of at least 11 from a wild-type reference sequence (‘CCTCACAGTTGCCAT’ – located between the two anchors). The fraction of HDR reads compared to the total read count was then used to calculate the HDR rate.

### Nanopore sequencing

Primer sequences used for Oxford Nanopore Technologies sequencing (ONT-seq) are listed in Supplementary Table 5. In total, 500 ng of gDNA was amplified using NEBNext High-Fidelity 2× PCR Master Mix and locus-specific primers containing 5′ tail Nanopore adapter sequences to yield PCR fragments ranging from 3.8 kb to 5.9 kb. The PCR cycling conditions included an initial denaturation at 98 °C for 30 s, followed by 25 cycles of denaturation at 98 °C for 10 s, annealing at 60 °C for 30 s, extension at 72 °C for 3 min and a final extension at 72 °C for 3 min. PCR products were purified using 0.8× homemade SPRI beads and quantified using Quant-iT dsDNA Assay Kits (Thermo Fisher Scientific) and VICTOR Nivo Microplate Reader (PerkinElmer). Then, 10 ng of PCR products was used for barcoding PCR using NEBNext High-Fidelity 2× PCR Master Mix and primers from PCR Barcoding Expansion 1–96 kit (ONT, EXP-PBC096). The PCR cycling conditions included an initial denaturation at 98 °C for 30 s, followed by six cycles of denaturation at 98 °C for 10 s, annealing at 62 °C for 15 s, extension at 72 °C for 3 min and a final extension at 72 °C for 3 min. Products of barcoding PCR were purified and quantified as previously described. PCR products were again purified using 0.8× homemade SPRI beads and quantified. The PCR products were evenly pooled to form a library containing 1 µg of DNA in 49 µl of water. End-prep, adapter ligation and clean-up were performed following the manufacturer’s instructions. Short-fragment buffer was used for final washing steps. Sequencing was performed by loading 25–30 fmol of DNA in MinION flow cell of GridION or Mk1C device.

### Long-read sequencing data analysis

ONT-seq of PCR amplicons was performed by the Functional Genomics Center Zürich (FGCZ) and provided as demultiplexed FASTQ data files.

FASTQ files were pre-processed using three consecutive rounds of Cutadapt (version 4.6). In Cutadapt round 1, reads were filtered and trimmed for intact end-to-end amplicons using the complete stubbing primer sequences for ONT-seq. In Cutadapt round 2, the reads were further filtered for amplicons specific to the genomic locus of interest using 30-bp gDNA sequences immediately following the round 1 primer binding sites. In Cutadapt round 3, amplicons larger than the unedited amplicon were discarded; a +20-bp size buffer was allowed to accommodate for innate errors in ONT-seq data.

As a quality control step, the surviving reads were aligned to the human genome (*GRCh38*) using minimap2 (version 2.24-r1122), and genome-wide coverage was calculated as counts per million (CPM) in 10-kbp bins using deepTools (version 3.5.4). In properly configured Cutadapt workflows, the majority (>95%) of reads after Cutadapt round 3 will align to the target genomic region for amplicon ONT-seq in unedited cells.

Indel rates and sizes were calculated from those reads passing all rounds of Cutadapt processing. Read lengths were extracted for all reads (bioawk, version 1.0) and counted. For each read length count, the fraction of total reads of greater length was calculated and plotted.

A summary of reads passing each Cutadapt processing step and genome alignment regions are provided in Supplementary Table 6. DNA sequences used in Cutadapt round 1 and round 2 are provided in Supplementary Table 7. The bash script and example input files used to summarize amplicon ONT-seq data are available online (https://github.com/cornlab/summarizeOntDeletions).

### scRNA-seq

Cells were sequenced using Chromium Controller (Firmware version 4.0) and Chromium Next GEM Single Cell 3′ Reagent Kits version 3.1 (10x Genomics) according to the manufacturer’s specifications. Libraries were sequenced by the FGCZ. Doublets, empty droplets and unhealthy cells were filtered out with Seurat (version 5.1.0) using number of reads per cell and percentage of mitochondrial reads. The inferCNV package (version 1.12.0) of the Trinity CTAT Project (https://github.com/broadinstitute/inferCNV) was then used to infer copy number variations. In brief, gene expression measurements of unedited cells were subtracted from those of edited cells using base R and dplyr (version 1.1.4). The edited cells were then clustered based on their residual gene expression levels up to the target site (chr12:6,538,000). Subsequently, these data were summarized in a heatmap focusing on chromosome 12 using the packages ggplot2 (version 3.5.1) and ComplexHeatmap (version 2.20.0). Additionally, the mean residual gene expression of genes spanning from the start of chromosome 12 to the target site was calculated and compared to the mean residual gene expression of the following 7 Mb (chr12:6,538,000–13,638,000).

### ddPCR

ddPCR analysis was conducted using the QX200 ddPCR System and QuantaSoft software version 1.7.4.0917 (Bio-Rad) according to the manufacturer’s instructions.

For VCN quantification of the FIRE reporter in the K-562 cell line, ddPCR assays were designed to target the U5-Psi region of the integrated lentiviral sequence and RPP30 for normalization. Droplets were generated using the QX200 AutoDG Droplet Generator (Bio-Rad) from a reaction mixture containing 50 ng of gDNA, 1× ddPCR Supermix for Probes (No dUTP) (Bio-Rad, 186-3024), 1× ddPCR assays (900 nM of each forward and reverse primer, 250 nM probe; Bio-Rad) and 10 U µg^−1^ DNA of the HaeIII restriction enzyme (NEB, R0108S) cutting outside of the amplicons. Generated droplets were then subjected to thermal cycling (10 min at 95 °C, 40 cycles of 30 s at 94 °C and 1 min at 60 °C, 10 min at 98 °C).

For copy number quantification in edited K-562 cells, ddPCR assays were designed to target the eGFP cassette, *KMT2C*, and an intergenic region (chr3:46,270,956–46,271,086) for normalization. In brief, 400–1,700 ng of gDNA was digested by HindIII-HF (20 U per reaction) and 1× rCutSmart buffer (NEB, R3104S and B6004S), followed by a 3-h incubation at 37 °C. The digested DNA was five-fold diluted in a buffer containing 2 ng μl^−1^ sheared salmon sperm DNA (Invitrogen, AM9680) and 0.05% Pluronic F-68 non-ionic surfactant (Gibco). Droplets were generated as described above but using the QX200 Droplet Generator (Bio-Rad) and 20 μl of diluted solution of digested gDNA. Generated droplets were then subjected to thermal cycling (10 min at 95 °C, 40 cycles of 30 s at 94 °C and 1 min at 57 °C, 10 min at 98 °C). Measurement and quantification were conducted using a QX200 Droplet Reader and QuantaSoft software (Bio-Rad).

### Sanger sequencing

Primer sequences used for Sanger sequencing are listed in Supplementary Table 5. In brief, a 599-bp region surrounding the target site of the FIRE reporter system from edited K-562 cells was amplified using NEBNext High-Fidelity 2× PCR Master Mix. The PCR products were purified using 1.8× SPRI beads. Subsequently, 1 µl of the purified PCR products underwent analysis using an Agilent 2200 TapeStation and TapeStation software version 5.1, along with D1000 ScreenTape and D1000 reagents, according to the manufacturer’s protocol. The purified samples were then subjected to Sanger sequencing (Microsynth AG), and the resulting traces were deconvoluted and analyzed using ICE^40^.

### CAST-seq

Primer sequences used for CAST-seq are listed in Supplementary Table 5. CAST-seq analyses were performed as previously described, with a few modifications^25^. In total, 200–220 ng of gDNA was used as input material for each technical replicate. Libraries were prepared using the NEBNext Ultra II FS DNA Library Prep Kit for Illumina (NEB, E7805L). Enzymatic fragmentation of the gDNA was aimed at an average length of 500–700 bp. CAST-seq libraries were sequenced on a NextSeq 2000 using 2 × 150-bp paired-end sequencing. For each sample, two technical replicates were run and analyzed, except for RPE-1 p53^+/+^ edited at CCR5 (*n* = 1). When two replicates were performed, only sites that were present in both technical replicates and significant in at least one replicate are shown in the circos plots and in Supplementary Table 2. For sites under investigation, the spacer sequence of the sgRNA was aligned in a window of ±400 bp around the most covered regions for each site. Sites were labeled as OMT if any of the two *P* values reached the cutoff of 0.005 (ref. ^26^).

### Statistical analysis

GraphPad Prism 10 software was used for statistical analysis. Results are presented as mean ± s.d. or as the mean only when there were fewer than three replicates.

### Reporting summary

Further information on research design is available in the Nature Portfolio Reporting Summary linked to this article.

## Online content

Any methods, additional references, Nature Portfolio reporting summaries, source data, extended data, supplementary information, acknowledgements, peer review information; details of author contributions and competing interests; and statements of data and code availability are available at 10.1038/s41587-024-02488-6.

## Supplementary information

Supplementary Tables 1–7

Reporting Summary

## Data Availability

Sequencing data for short-read, long-read and CAST sequencing are available in the Sequence Read Archive under BioProject PRJNA1167903 (ref. ^41^). GRCh38 was downloaded from GenBank. All raw data supporting the findings of this study are available from the corresponding author upon reasonable request.

## Code availability

The code used for analysis of long-read sequencing data is available on GitHub (https://github.com/cornlab/summarizeOntDeletions). The code for CAST-seq analysis is available on GitHub (https://github.com/AG-Boerries/CAST-Seq)^42^.

## References

[1] Scully, R., Panday, A., Elango, R. & Willis, N. A. DNA double-strand break repair-pathway choice in somatic mammalian cells. *Nat. Rev. Mol. Cell Biol.* **20**, 698–714 (2019). 31263220 10.1038/s41580-019-0152-0 PMC7315405

[2] Yeh, C. D., Richardson, C. D. & Corn, J. E. Advances in genome editing through control of DNA repair pathways. *Nat. Cell Biol.* **21**, 1468–1478 (2019). 31792376 10.1038/s41556-019-0425-z

[3] Frangoul, H. et al. CRISPR–Cas9 gene editing for sickle cell disease and β-thalassemia. *N. Engl. J. Med.* **384**, 252–260 (2021). 33283989 10.1056/NEJMoa2031054

[4] Shin, J. J. et al. Controlled cycling and quiescence enables efficient HDR in engraftment-enriched adult hematopoietic stem and progenitor cells. *Cell Rep.* **32**, 108093 (2020). 10.1016/j.celrep.2020.108093 PMC7487781 32877675

[5] Lomova, A. et al. Improving gene editing outcomes in human hematopoietic stem and progenitor cells by temporal control of DNA repair. *Stem Cells* **37**, 284–294 (2019). 30372555 10.1002/stem.2935 PMC6368869

[6] Charpentier, M. et al. CtIP fusion to Cas9 enhances transgene integration by homology-dependent repair. *Nat. Commun.* **9**, 1133 (2018). 29556040 10.1038/s41467-018-03475-7 PMC5859065

[7] Aird, E. J., Lovendahl, K. N., St Martin, A., Harris, R. S. & Gordon, W. R. Increasing Cas9-mediated homology-directed repair efficiency through covalent tethering of DNA repair template. *Commun. Biol.* **1**, 54 (2018). 30271937 10.1038/s42003-018-0054-2 PMC6123678

[8] Möller, L. et al. Recursive editing improves homology-directed repair through retargeting of undesired outcomes. *Nat. Commun.* **13**, 4550 (2022). 35931681 10.1038/s41467-022-31944-7 PMC9356142

[9] Jayavaradhan, R. et al. CRISPR–Cas9 fusion to dominant-negative 53BP1 enhances HDR and inhibits NHEJ specifically at Cas9 target sites. *Nat. Commun.* **10**, 2866 (2019). 31253785 10.1038/s41467-019-10735-7 PMC6598984

[10] Wienert, B. et al. Timed inhibition of CDC7 increases CRISPR–Cas9 mediated templated repair. *Nat. Commun.* **11**, 2109 (2020). 32355159 10.1038/s41467-020-15845-1 PMC7193628

[11] Carusillo, A. et al. A novel Cas9 fusion protein promotes targeted genome editing with reduced mutational burden in primary human cells. *Nucleic Acids Res.* **51**, 4660–4673 (2023). 37070192 10.1093/nar/gkad255 PMC10201422

[12] Wen, W. et al. Effective control of large deletions after double-strand breaks by homology-directed repair and dsODN insertion. *Genome Biol.* **22**, 236 (2021). 34416913 10.1186/s13059-021-02462-4 PMC8377869

[13] Robert, F., Barbeau, M., Éthier, S., Dostie, J. & Pelletier, J. Pharmacological inhibition of DNA-PK stimulates Cas9-mediated genome editing. *Genome Med.* **7**, 93 (2015). 26307031 10.1186/s13073-015-0215-6 PMC4550049

[14] Riesenberg, S. et al. Simultaneous precise editing of multiple genes in human cells. *Nucleic Acids Res.* **47**, e116 (2019). 31392986 10.1093/nar/gkz669 PMC6821318

[15] Riesenberg, S. & Maricic, T. Targeting repair pathways with small molecules increases precise genome editing in pluripotent stem cells. *Nat. Commun.* **9**, 2164 (2018). 29867139 10.1038/s41467-018-04609-7 PMC5986859

[16] Fu, Y.-W. et al. Dynamics and competition of CRISPR–Cas9 ribonucleoproteins and AAV donor-mediated NHEJ, MMEJ and HDR editing. *Nucleic Acids Res.* **49**, 969–985 (2021). 33398341 10.1093/nar/gkaa1251 PMC7826255

[17] Wimberger, S. et al. Simultaneous inhibition of DNA-PK and Polθ improves integration efficiency and precision of genome editing. *Nat. Commun.* **14**, 4761 (2023). 37580318 10.1038/s41467-023-40344-4 PMC10425386

[18] Selvaraj, S. et al. High-efficiency transgene integration by homology-directed repair in human primary cells using DNA-PKcs inhibition. *Nat. Biotechnol* **42**, 731–744 (2024). 10.1038/s41587-023-01888-4 37537500

[19] Cloarec-Ung, F.-M. et al. Near-perfect precise on-target editing of human hematopoietic stem and progenitor cells. *eLife*10.7554/eLife.91288 (2023). 10.7554/eLife.91288 PMC11147503 38829685

[20] Cullot, G. et al. CRISPR–Cas9 genome editing induces megabase-scale chromosomal truncations. *Nat. Commun.* **10**, 1136 (2019). 30850590 10.1038/s41467-019-09006-2 PMC6408493

[21] Boutin, J. et al. CRISPR–Cas9 globin editing can induce megabase-scale copy-neutral losses of heterozygosity in hematopoietic cells. *Nat. Commun.* **12**, 4922 (2021). 34389729 10.1038/s41467-021-25190-6 PMC8363739

[22] Alanis-Lobato, G. et al. Frequent loss of heterozygosity in CRISPR–Cas9-edited early human embryos. *Proc. Natl Acad. Sci. USA* **118**, e2004832117 (2021). 34050011 10.1073/pnas.2004832117 PMC8179174

[23] Tsuchida, C. A. et al. Mitigation of chromosome loss in clinical CRISPR–Cas9-engineered T cells. *Cell* **186**, 4567–4582 (2023). 37794590 10.1016/j.cell.2023.08.041 PMC10664023

[24] Wang, J., Sadeghi, C. A. & Frock, R. L. DNA-PKcs suppresses illegitimate chromosome rearrangements. *Nucleic Acids Res.* **52**, 5048–5066 (2024). 38412274 10.1093/nar/gkae140 PMC11109964

[25] Turchiano, G. et al. Quantitative evaluation of chromosomal rearrangements in gene-edited human stem cells by CAST-Seq. *Cell Stem Cell* **28**, 1136–1147 (2021). 33626327 10.1016/j.stem.2021.02.002

[26] Klermund, J. et al. On- and off-target effects of paired CRISPR–Cas nickase in primary human cells. *Mol. Ther.* **32**, 1298–1310 (2024). 38459694 10.1016/j.ymthe.2024.03.006 PMC11081867

[27] Schimmel, J. et al. Modulating mutational outcomes and improving precise gene editing at CRISPR–Cas9-induced breaks by chemical inhibition of end-joining pathways. *Cell Rep.* **42**, 112019 (2023). 36701230 10.1016/j.celrep.2023.112019

[28] Black, S. J. et al. Molecular basis of microhomology-mediated end-joining by purified full-length Polθ. *Nat. Commun.* **10**, 4423 (2019). 31562312 10.1038/s41467-019-12272-9 PMC6764996

[29] Kosicki, M., Tomberg, K. & Bradley, A. Repair of double-strand breaks induced by CRISPR–Cas9 leads to large deletions and complex rearrangements. *Nat. Biotechnol.* **36**, 765–771 (2018). 30010673 10.1038/nbt.4192 PMC6390938

[30] Fiumara, M. et al. Genotoxic effects of base and prime editing in human hematopoietic stem cells. *Nat. Biotechnol.* **42**, 877–891 (2024). 10.1038/s41587-023-01915-4 PMC11180610 37679541

[31] Huang, M. E. et al. C-to-G editing generates double-strand breaks causing deletion, transversion and translocation. *Nat. Cell Biol.* **26**, 294–304 (2024). 38263276 10.1038/s41556-023-01342-2

[32] Chen, P. J. et al. Enhanced prime editing systems by manipulating cellular determinants of editing outcomes. *Cell* **184**, 5635–5652 (2021). 34653350 10.1016/j.cell.2021.09.018 PMC8584034

[33] Ferreira da Silva, J. et al. Prime editing efficiency and fidelity are enhanced in the absence of mismatch repair. *Nat. Commun.* **13**, 760 (2022). 35140211 10.1038/s41467-022-28442-1 PMC8828784

[34] Chiang, T.-W. W., le Sage, C., Larrieu, D., Demir, M. & Jackson, S. P. CRISPR–Cas9^D10A^ nickase-based genotypic and phenotypic screening to enhance genome editing. *Sci. Rep.* **6**, 24356 (2016). 27079678 10.1038/srep24356 PMC4832145

[35] Throm, R. E. et al. Efficient construction of producer cell lines for a SIN lentiviral vector for SCID-X1 gene therapy by concatemeric array transfection. *Blood* **113**, 5104–5110 (2009). 19286997 10.1182/blood-2008-11-191049 PMC2686181

[36] Wielgosz, M. M. et al. Generation of a lentiviral vector producer cell clone for human Wiskott–Aldrich syndrome gene therapy. *Mol. Ther. Methods Clin. Dev.* **2**, 14063 (2015). 26052531 10.1038/mtm.2014.63 PMC4449020

[37] Anders, C. & Jinek, M. In vitro enzymology of Cas9. *Methods Enzymol.* **546**, 1–20 (2014). 25398333 10.1016/B978-0-12-801185-0.00001-5 PMC5074358

[38] Wienert, B., Shin, J., Zelin, E., Pestal, K. & Corn, J. E. In vitro-transcribed guide RNAs trigger an innate immune response via the RIG-I pathway. *PLoS Biol.* **16**, e2005840 (2018). 30011268 10.1371/journal.pbio.2005840 PMC6049001

[39] Clement, K. et al. CRISPResso2 provides accurate and rapid genome editing sequence analysis. *Nat. Biotechnol.* **37**, 224–226 (2019). 30809026 10.1038/s41587-019-0032-3 PMC6533916

[40] Conant, D. et al. Inference of CRISPR edits from Sanger trace data. *CRISPR J.* **5**, 123–130 (2022). 35119294 10.1089/crispr.2021.0113

[41] Cullot, G. et al. Genome editing with the HDR-enhancing DNA-PKcs inhibitor AZD7648 causes large-scale genomic alterations. https://www.ncbi.nlm.nih.gov/sra/?term=PRJNA1167903 (2024). 10.1038/s41587-024-02488-6 PMC12611759 39604565

[42] Cullot, G. et al. Genome editing with the HDR-enhancing DNA-PKcs inhibitor AZD7648 causes large-scale genomic alterations. https://github.com/AG-Boerries/CAST-Seq (2024). 10.1038/s41587-024-02488-6 PMC12611759 39604565

