# Supplementary material for: Genome editing with the HDR-enhancing DNA-PKcs inhibitor AZD7648 causes large-scale genomic alterations
Source: Nat Biotechnol. 2024 Nov 27;43(11):1778–82. doi: 10.1038/s41587-024-02488-6 (PMC12611759; doi:10.1038/s41587-024-02488-6)
Supplement: Supplementary file 1 — Supplementary Tables 1–7 [file 41587_2024_2488_MOESM1_ESM.pdf]

nature biotechnology

Supplementary information https://doi.org/10.1038/s41587-024-02488-6

Genome editing with the HDR-enhancing DNA-PKcs inhibitor AZD7648 causes large-scale genomic alterations

In the format provided by the authors and unedited

Table of contents

Supplemental Table 1 – Editing reagent sequences.
Supplemental Table 2 – Translocations identified with CAST-seq.
Supplemental Table 3 – Media composition for upper airway organoid culture.
Supplemental Table 4 – Transfection conditions.
Supplemental Table 5 – Oligonucleotide sequences.
Supplemental Table 6 – Number of reads at each step of long-read sequencing analysis.
Supplemental Table 7 – Reference sequence used for long-read sequencing analysis.

Supplemental Table 1

Cell type	Target	Cas9	sgRNA		ssODN			
			Origin	Sequence	Origin	Sequence	Length (nt)	Mutation type
K-562	HBB	Home-made SpCas9-NLS	Alt-R CRISPR-Cas9 sgRNA (IDT)	CTTGCCCCACAGGGCAGTAA	Alt-R HDR Donor oligo (IDT)	TCAGGGCAGAGCCATCTATTGCTTACATTTGCTTCTGACACAACTGTGTTCACTAGC<br>AACCTCAAACAGACACCATGGTGCACCTGACTCCTGTAGAGAAGTCTGCGGTTACTG<br>CCCTGTGGGGCAAGGTGAACGTGGATGAAGTTGGTGGTGAGGCCCTGGGCA*G*G*T	171	substitution (12nt)
K-562	CCR5	Home-made SpCas9-NLS	Alt-R CRISPR-Cas9 sgRNA (IDT)	GGCAGCATAGTGAGCCCAGA	Alt-R HDR Donor oligo (IDT)	TGAAGAGCATGACTGACATCTACCTGCTCAACCTGGCCATCTCTGACCTGTTTTTCC<br>TTCTTACTGTCCCtTTtTGGGCaCAtTATGCTGCCGCCCAGTGGGACTTTGGAAATA<br>CAATGTGTCAACTCTTGACAGGGCTCTATTTTATAG	150	substitution (4 nt)
K-562	HBD	Home-made SpCas9-NLS	Alt-R CRISPR-Cas9 sgRNA (IDT)	CAGGACCAGCATAAAAGGCA	Alt-R HDR Donor oligo (IDT)	GGAATAGTGGAATGAAGGTTCATTTTTCATTCTCACAAACTAATGAAACCCTGCTTA<br>TCTTAAACCAACCTGCTCACCACACCCTGGAGCAGGGAGGGCAGGAGCCAGGGCTGG<br>GCATAAAAGGCAGGGCAGAGTCGACTGTTGCTTACACTTTCTTCTGACATAACAGTG<br>TTCACTAGCAACCTCAAACAGAC	194	insertion (15 nt) + substitution (2nt)
K-562	TRAC	Home-made SpCas9-NLS	CRISPRevolution sgRNA EZ Kit (Synthego)	TCAGGGTTCTGGATATCTGT	Microsynth	AAATGAGATCATGTCCTAACCCTGATCCTCTTGTCCCACACATGATATCCAGAACCC<br>TGACCCTGCCGTGTACCAGCTGAGAG	83	insertion (3 nt)
K-562	GAPDH	Home-made SpCas9-NLS	CRISPRevolution sgRNA EZ Kit (Synthego)	GGGTCTACATGGCAACTGTG	Alt-R HDR Donor oligo (IDT)	GAAGAGAGAGACCCTCACTGCTGGGGAGTCCCTGCCACACTCAGTCCCCCACCACAC<br>TGAATCTCCCCTTGTDBDBHVVHDDBVGTAGACCCCTTGAAGAGGGGAGGGGCCTAG<br>GGAGCCGCACCTTGTCATGTACCATCAATAAAGTAC	150	substitution barcode (12nt)
K-562	AAVS1	Home-made SpCas9-NLS	CRISPRevolution sgRNA EZ Kit (Synthego)	TCCCTAGTGGCCCCACTGTG	Microsynth	CTCTGGTTCTGGGTACTTTTATCTGTCCCCTCCACCCCACGATAGTGGGGCCACTAG<br>GGACAGGATTGGTGACAGAAAAGCCC	83	insertion (3 nt)
K-562 FIRE reporter	FIRE reporter	Home-made SpCas9-NLS	CRISPRevolution sgRNA EZ Kit (Synthego)	CATGGAGGGCTCCATGTTCG	GenExact™ Single-stranded DNA	GCTCTATATAAGCAGAGCTCGTTTAGTGAACCGTCAGATCGGCGCGCCAATTCAAGC<br>GACAAGACAAGGGCAGCCGCCACCATGGTCAGCAAGGGCGAGGCCGTCATCAAGGAG<br>TTCATGCGGTTCAAGGTGCACATGGAGGGCTCCATGAACGGTCACGAGTTCGAGATC<br>GAGGGCGAAGGCGAGGGCAGGCCCTACGAGGGCACCCAGACCGCCAAGCTGAAGGTG<br>ACAAAGGGCGGCCCCCTGCCCTTCTCCTGGGACATCCTGTCCCCTCAGTTCATGTAC<br>GGCTCCAGGGCCTTCATCAAGCACCCCGCCGACATCCCCGACTACTACAAGCAGTCC<br>TTCCCCGAGGGCTTCAAGTGGGAGAGGGTGATGAACTTCGAGGACGGCGGCGCCGTC<br>ACCGTCACCCAGGACACCTCCCTGGAGGACGGCACCCTCATCTACAAGGTCAAGCTG<br>CGGGGCACCAACTTCCCTCCAGACGGCCCCGTCATGCAGAAGAAAACAATGGGCTGG<br>GAGGCCTCCACCGAG	528	insertion (228 nt)
K-562 eGFP	SMURF1	Home-made SpCas9-NLS	Alt-R CRISPR-Cas9 sgRNA (IDT)	TCTATCACGAGCCTAACCCG	Alt-R HDR Donor oligo (IDT)	TTCCTCTTGAAGGTGAGGAAGTGAAAACTACAGACCTCCCCCGGACAGCCCACTCTC<br>TATCACGAGCCTAACGGTACCCCGCGGGAGGCGGAAGAGACATCCATTCGAGAACTG<br>AAGCGGCCTCCGGGATGAGGTCAGAGGCCCCACCTG	150	insertion (6 nt)
K-562 eGFP	IFT22	Home-made SpCas9-NLS	Alt-R CRISPR-Cas9 sgRNA (IDT)	GTTGTATCATTGGGCTGCGA	Alt-R HDR Donor oligo (IDT)	CAAATTTGGATTTCCTGGCCCCAGAACTTCCCAAAGACCTGTAAAATGGAGGGATTT<br>ACCACCTCACATATGTCCAGTTAAACAGTTTGTGGACTTGTAACCGTCGGGTACCCA<br>GCCCAATGATACAACAGTAGTTTAATCACGTGTATT	150	insertion (6 nt)
RPE-1 +/- p53	HBB	Home-made SpCas9-NLS	Alt-R CRISPR-Cas9 sgRNA (IDT)	CTTGCCCCACAGGGCAGTAA	Alt-R HDR Donor oligo (IDT)	TCAGGGCAGAGCCATCTATTGCTTACATTTGCTTCTGACACAACTGTGTTCACTAGC<br>AACCTCAAACAGACACCATGGTGCACCTGACTCCTGTAGAGAAGTCTGCGGTTACTG<br>CCCTGTGGGGCAAGGTGAACGTGGATGAAGTTGGTGGTGAGGCCCTGGGCA*G*G*T	171	substitution (12nt)
RPE-1 +/- p53	CCR5	Home-made SpCas9-NLS	Alt-R CRISPR-Cas9 sgRNA (IDT)	GGCAGCATAGTGAGCCCAGA	Alt-R HDR Donor oligo (IDT)	TGAAGAGCATGACTGACATCTACCTGCTCAACCTGGCCATCTCTGACCTGTTTTTCC<br>TTCTTACTGTCCCtTTtTGGGCaCAtTATGCTGCCGCCCAGTGGGACTTTGGAAATA<br>CAATGTGTCAACTCTTGACAGGGCTCTATTTTATAG	150	substitution (4 nt)
RPE-1 +/- p53	HBD	Home-made SpCas9-NLS	Alt-R CRISPR-Cas9 sgRNA (IDT)	CAGGACCAGCATAAAAGGCA	Alt-R HDR Donor oligo (IDT)	GGAATAGTGGAATGAAGGTTCATTTTTCATTCTCACAAACTAATGAAACCCTGCTTA<br>TCTTAAACCAACCTGCTCACCACACCCTGGAGCAGGGAGGGCAGGAGCCAGGGCTGG<br>GCATAAAAGGCAGGGCAGAGTCGACTGTTGCTTACACTTTCTTCTGACATAACAGTG<br>TTCACTAGCAACCTCAAACAGAC	194	insertion (15 nt) + substitution (2nt)
RPE-1 +/- p53	TRAC	Home-made SpCas9-NLS	CRISPRevolution sgRNA EZ Kit (Synthego)	TCAGGGTTCTGGATATCTGT	Microsynth	AAATGAGATCATGTCCTAACCCTGATCCTCTTGTCCCACACATGATATCCAGAACCC<br>TGACCCTGCCGTGTACCAGCTGAGAG	83	insertion (3 nt)
RPE-1 +/- p53	GAPDH	Home-made SpCas9-NLS	CRISPRevolution sgRNA EZ Kit (Synthego)	GGGTCTACATGGCAACTGTG	Alt-R HDR Donor oligo (IDT)	GAAGAGAGAGACCCTCACTGCTGGGGAGTCCCTGCCACACTCAGTCCCCCACCACAC<br>TGAATCTCCCCTTGTDBDBHVVHDDBVGTAGACCCCTTGAAGAGGGGAGGGGCCTAG<br>GGAGCCGCACCTTGTCATGTACCATCAATAAAGTAC	150	substitution barcode (12nt)
RPE-1 +/- p53	AAVS1	Home-made SpCas9-NLS	In vitro transcription	TCCCTAGTGGCCCCACTGTG	Microsynth	CTCTGGTTCTGGGTACTTTTATCTGTCCCCTCCACCCCACGATAGTGGGGCCACTAG<br>GGACAGGATTGGTGACAGAAAAGCCC	83	insertion (3 nt)
HSPC	HBB	lt-R™ S.p. Cas9 Nuclease V3 (IDT	Alt-R CRISPR-Cas9 sgRNA (IDT)	CTTGCCCCACAGGGCAGTAA	Alt-R HDR Donor oligo (IDT)	TCAGGGCAGAGCCATCTATTGCTTACATTTGCTTCTGACACAACTGTGTTCACTAGC<br>AACCTCAAACAGACACCATGGTGCACCTGACTCCTGTAGAGAAGTCTGCGGTTACTG<br>CCCTGTGGGGCAAGGTGAACGTGGATGAAGTTGGTGGTGAGGCCCTGGGCA*G*G*T	171	substitution (12nt)
HSPC	CCR5	lt-R™ S.p. Cas9 Nuclease V3 (IDT	Alt-R CRISPR-Cas9 sgRNA (IDT)	GGCAGCATAGTGAGCCCAGA	Alt-R HDR Donor oligo (IDT)	TGAAGAGCATGACTGACATCTACCTGCTCAACCTGGCCATCTCTGACCTGTTTTTCC<br>TTCTTACTGTCCCtTTtTGGGCaCAtTATGCTGCCGCCCAGTGGGACTTTGGAAATA<br>CAATGTGTCAACTCTTGACAGGGCTCTATTTTATAG	150	substitution (4 nt)
HSPC	GAPDH	lt-R™ S.p. Cas9 Nuclease V3 (IDT	CRISPRevolution sgRNA EZ Kit (Synthego)	GGGTCTACATGGCAACTGTG	Alt-R HDR Donor oligo (IDT)	CAAGAGGAAGAGAGAGACCCTCACTGCTGGGGAGTCCCTGCCACACTCAGTCCCCCA<br>CCACACTGAATCTCCCCTTGTACACTGGATTCAGTAGACCCCTTGAAGAGGGGAGGG<br>GCCTAGGGAGCCGCACCTTGTCATGTACCATCAATAAAGTACCCTGTGCTC	150	substitution (12nt)
HSPC (scRNA-seq)	GAPDH	Home-made SpCas9-NLS	CRISPReverseolution sgRNA EZ Kit (Synthego)	GGGTCTACATGGCAACTGTG	Alt-R HDR Donor oligo (IDT)	CAAGAGGAAGAGAGAGACCCTCACTGCTGGGGAGTCCCTGCCACACTCAGTCCCCCA<br>CCACACTGAATCTCCCCTTGTDBDBHVVHDDBVGTAGACCCCTTGAAGAGGGGAGGG<br>GCCTAGGGAGCCGCACCTTGTCATGTACCATCAATAAAGTACCCTGTGCTC	150	substitution barcode (12nt)
Upper airway organoid	GAPDH	Home-made SpCas9-NLS	CRISPRevolution sgRNA EZ Kit (Synthego)	GGGTCTACATGGCAACTGTG	Alt-R HDR Donor oligo (IDT)	CAAGAGGAAGAGAGAGACCCTCACTGCTGGGGAGTCCCTGCCACACTCAGTCCCCCA<br>CCACACTGAATCTCCCCTTGTDBDBHVVHDDBVGTAGACCCCTTGAAGAGGGGAGGG<br>GCCTAGGGAGCCGCACCTTGTCATGTACCATCAATAAAGTACCCTGTGCTC	150	substitution barcode (12nt)

Supplemental Table 2

Target	Cell line	Condition	Total read (rep1)	Total read (rep2)	Category	chr	start	end	reads (rep1)	reads (rep2)	hits (rep1)	hits (rep2)	Annotation	Closest element	Putative target site	Score
HBB	HSCs	Cas9 + ssODN	414'567	596'827	ON	chr11	5'225'112	5'228'549	4'892	11'102	117	122	Promoter (<=1kb)	HBB	CTTGCCCCACAGGGCAGTAACGG	20.5
					Deletion to HBD	chr11	5'234'125	5'234'655	8'946	14'635	32	41	5' UTR	HBD	TTTGCCCCACAGGGCATTGACAG	14.5
					OMT1	chr9	101'833'332	101'833'859	367	465	6	5	Distal Intergenic	GRIN3A	CTTCAGCCCCACAGGGCAGTAAGGG	17.5
		Cas9 + ssODN + AZD7648	482'138	315'288	ON	chr11	5'224'021	5'234'941	60'684	29'959	129	106	Promoter (<=1kb)	HBB	CTTGCCCCACAGGGCAGTAACGG	20.5
					OMT1	chr9	101'832'145	101'834'365	5'803	4'362	67	56	Distal Intergenic	GRIN3A	CTTCAGCCCCACAGGGCAGTAAGGG	17.5
					OMT2	chr12	124'319'031	124'319'545	2'337	1'082	12	13	Distal Intergenic	MIR6880	CT-GCCCCACAGGGCAGCAAAGG	15.5
					OMT3	chr17	78'243'303	78'243'814	582	241	7	3	Distal Intergenic	LOC105371910	CTTGCCCCAGACAGGGCAGTAAGGG	17.5
					OMT4	chrX	75'786'171	75'786'671	123	20	2	1	Promoter (<=1kb)	MAGEE2	GTGGCCCCACAGGGCAGGAATGG	14.5
CCR5	HSCs	Cas9 + ssODN	3'807'449	3'723'426	ON	chr3	46'363'453	46'378'217	4'352	5'233	195	176	Promoter (<=1kb)	CCR5	GGCAGCATAGTGAGCCCAGAAGG	20.5
					OMT1	chr11	16'159'958	16'161'306	155	257	23	37	Intron (intron 5 of 14)	SOX6	AGCAGCACAGTGAGCCCAGGGGG	14.5
		Cas9 + ssODN + AZD7648	3'944'929	5'052'178	ON	chr3	46'370'917	46'378'088	2'103	2'669	123	142	Promoter (<=1kb)	CCR5	GGCAGCATAGTGAGCCCAGAAGG	20.5
					OMT1	chr11	16'157'300	16'162'682	1'051	748	73	68	Intron (intron 5 of 14)	SOX6	AGCAGCACAGTGAGCCCAGGGGG	14.5
					OMT2	chr14	105'852'906	105'853'419	169	93	6	6	Intron (intron 4 of 5)	MIR4539	GACTAAGCATA-TGGGCCCAGAGGG	10.5
					OMT3	chr3	46'357'499	46'358'069	39	44	5	5	5' UTR	CCR2	TGCAGCAGAGTGAGCCCACAATG	13.5
					OMT4	chr14	55'463'094	55'463'599	1	13	1	3	Distal Intergenic	TBPL2	AACAGCATAGTGAACCCAGGAGG	12.5
					OMT5	chr17	44'077'075	44'077'576	16	74	1	2	3' UTR	HDAC5	GTCAGCAAAGGGAGCCCAGAAGG	14.5
					OMT6	chr14	91'323'991	91'324'491	17	19	1	1	Intron (intron 12 of 29)	CCDC88C	GGCAGCAGAGCAAGCCCAGGAGG	12.5
GAPDH	HSCs	Cas9 + ssODN	1'370'744	1'031'714	ON	chr12	6'533'860	6'540'931	654	352	40	25	Promoter (<=1kb)	GAPDH	GGGTCTACATGGCAACTGTGAGG	20.5
		Cas9 + ssODN + AZD7648	1'101'134	1'707'332	ON	chr12	6'533'349	6'548'412	984	1'142	83	82	Promoter (<=1kb)	GAPDH	GGGTCTACATGGCAACTGTGAGG	20.5
					OMT1	chr12	53'219'201	53'219'709	223	174	13	6	Intron (intron 2 of 8)	RARG	AGGTCCACATGGC--CTGTGAGG	11.5
					OMT2	chr10	124'473'266	124'473'799	84	33	8	8	Intron (intron 1 of 6)	LHPP	GAGGCCACATGGCAACTATGGGG	12.5
					OMT3	chr4	107'862'066	107'863'318	14	80	1	6	Intron (intron 2 of 2)	SGMS2	GCTCCTACATGGTAACTGTGAGG	12.5
					OMT4	chr1	110'612'401	110'612'905	17	60	2	4	Intron (intron 1 of 4)	KCNA2	GGGTCTCTATAGCAACTGTGGGG	14.5
					OMT5	chr12	726'027	726'542	29	4	4	1	Distal Intergenic	WNK1	AGGTCTGCATGGCA-CTGTGAAG	13.5
					OMT6	chr19	45'372'302	45'372'803	21	48	3	1	Promoter (1-2kb)	ERCC2	GGATGTCTAC-TGTCAACTGTGGGG	12.5
					OMT7	chr2	103'803'591	103'804'096	1	19	1	3	Distal Intergenic	LINC01965	GAGGCTACATAGCAACAGTGAGG	12.5
					OMT8	chr14	51'884'315	51'884'816	10	5	2	2	Intron (intron 2 of 3)	GNG2	CAGACCACATGGTAACTGTGAGG	10.5
					OMT9	chr3	126'818'682	126'819'183	49	11	2	1	Intron (intron 5 of 7)	CHCHD6	AGGCCTACGTGGCAACTGTGGAG	14.5
					OMT10	chr7	101'237'289	101'237'865	20	10	2	1	Promoter (<=1kb)	CLDN15	GCTTCTTCATGGCAACTGTGGGG	14.5

Target	Cell line	Condition	Total read (rep1)	Total read (rep2)	Category	chr	start	end	reads (rep1)	reads (rep2)	hits (rep1)	hits (rep2)	Annotation	Closest element	Putative target site	Score
HBB	RPE-1 p53-/-	Cas9 + ssODN	1'494'728	1'443'632	ON	chr11	5'221'187	5'242'723	174'049	150'105	518	548	Promoter (<=1kb)	HBB	CTTGCCCCACAGGGCAGTAACGG	20.5
					OMT1	chr9	101'833'341	101'833'859	1'475	349	5	4	Distal Intergenic	GRIN3A	CTTCAGCCCCACAGGGCAGTAAGGG	17.5
		Cas9 + ssODN + AZD7648	986'117	1'045'525	ON	chr11	5'220'728	5'235'876	234'492	133'564	434	405	Promoter (<=1kb)	HBB	CTTGCCCCACAGGGCAGTAACGG	20.5
					OMT1	chr9	101'831'953	101'835'418	2'780	9'955	22	37	Distal Intergenic	GRIN3A	CTTCAGCCCCACAGGGCAGTAAGGG	17.5
					OMT2	chr12	124'319'035	124'319'549	6	677	1	4	Distal Intergenic	MIR6880	CT-GCCCCACAGGGCAGCAAAGG	15.5
	RPE-1 p53+/+	Cas9 + ssODN	1'448'105	1'234'594	ON	chr11	5'219'668	5'236'813	31'162	25'314	227	269	Promoter (<=1kb)	HBB	CTTGCCCCACAGGGCAGTAACGG	20.5
		Cas9 + ssODN + AZD7648	1'052'897	985'094	ON	chr11	5'224'830	5'230'761	14'776	11'791	125	106	Promoter (<=1kb)	HBB	CTTGCCCCACAGGGCAGTAACGG	20.5
					OMT1	chr9	101'832'726	101'834'804	8'316	8'136	45	38	Distal Intergenic	GRIN3A	CTTCAGCCCCACAGGGCAGTAAGGG	17.5
					OMT2	chr12	124'319'037	124'319'541	310	1'066	2	5	Distal Intergenic	MIR6880	CT-GCCCCACAGGGCAGCAAAGG	15.5
					OMT3	chr11	126'115'573	126'116'073	287	548	2	1	Distal Intergenic	LOC105369591	ATTACCCCACAGGGCAGT--GGG	11.5
					OMT4	chr17	78'243'307	78'243'808	1	562	1	1	Distal Intergenic	LOC105371910	CTTGCCCCAGACAGGGCAGTAAGGG	17.5
CCR5	RPE-1 p53-/-	Cas9 + ssODN	1'156'349	1'116'787	ON	chr3	46'366'228	46'380'887	12'093	23'452	246	338	Promoter (<=1kb)	CCR5	GGCAGCATAGTGAGCCCAGAAGG	20.5
					OMT1	chr3	46'356'820	46'358'204	354	145	11	17	Promoter (2-3kb)	CCR2	TGCAGCAGAGTGAGCCCACAATG	13.5
		Cas9 + ssODN + AZD7648	2'126'782	1'865'952	ON	chr3	46'355'973	46'380'234	549'435	482'705	2583	2716	Promoter (<=1kb)	CCR5	GGCAGCATAGTGAGCCCAGAAGG	20.5
					OMT1 / HMT	chr14	55'461'620	55'465'727	2'500	12'787	15	51	Distal Intergenic	TBPL2	AACAGCATAGTGAACCCAGGAGG	12.5
					OMT2	chr11	16'160'153	16'161'309	3'270	2'771	16	13	Intron (intron 5 of 14)	SOX6	AGCAGCACAGTGAGCCCAGGGGG	14.5
					OMT3	chr13	95'195'150	95'196'765	1'703	914	2	6	Intron (intron 8 of 30)	ABCC4	GGAAGCA-AGTGAGCCCAGAAGG	15.5
					HMT	chr1	202'169'830	202'174'244	2'674	726	6	5	Promoter (1-2kb)	PTPRVP	–	-
	RPE-1 p53+/+	Cas9 + ssODN	1'403'331	-	ON	chr3	46'371'646	46'374'562	95	-	21	-	5' UTR	CCR5AS	GGCAGCATAGTGAGCCCAGAAGG	20.5
					HMT	chr22	30'843'750	30'844'253	45	-	3	-	Intron (intron 3 of 14)	OSBP2	–	-
					HMT	chr2	112'815'826	112'816'333	57	-	2	-	Distal Intergenic	LOC124907871	–	-
		Cas9 + ssODN + AZD7648	2'284'541	-	ON	chr3	46'370'946	46'376'271	37'222	-	359	-	Promoter (<=1kb)	CCR5	GGCAGCATAGTGAGCCCAGAAGG	20.5
					OMT1	chr14	55'463'090	55'463'603	4'341	-	29	-	Distal Intergenic	TBPL2	AACAGCATAGTGAACCCAGGAGG	12.5
					OMT2	chr11	16'160'505	16'162'844	1'805	-	30	-	Intron (intron 5 of 14)	SOX6	AGCAGCACAGTGAGCCCAGGGGG	14.5
					OMT3	chr13	95'195'159	95'195'667	1'247	-	10	-	Intron (intron 8 of 30)	ABCC4	GGAAGCA-AGTGAGCCCAGAAGG	15.5
					OMT4	chr10	77'610'687	77'611'191	493	-	8	-	Intron (intron 1 of 27)	KCNMA1	GGCA-CATCCAGAGAGCCCACACAG	10.5
					OMT5	chr18	44'884'270	44'884'770	162	-	4	-	Intron (intron 3 of 5)	SETBP1	GGCAGCATTGGGAGCCCAG-GGG	13.5
					OMT6	chr10	98'300'072	98'300'573	200	-	3	-	Distal Intergenic	LOXL4	GGCAG-ATAATGAGCCCAGA-GG	14
					OMT7	chr1	25'236'334	25'236'838	81	-	3	-	Distal Intergenic	SYF2	GG-AGCTTGCAGTGAGCCCAGATTG	11.5
					OMT8	chr17	43'575'622	43'576'126	453	-	2	-	Intron (intron 24 of 24)	DHX8	GTCAGCATGGTGAGCCCAGA-GG	15
					OMT9	chr3	73'656'082	73'656'590	432	-	2	-	Distal Intergenic	PDZRN3	TGCAGCATTGAGAGCCCAG-GGG	11.5
					OMT10	chr12	69'837'322	69'837'822	343	-	2	-	Intron (intron 4 of 4)	MYRFL	GGCAGGGAAGTGTGCTCCAGAAGG	10.5
					OMT11	chr5	57'804'159	57'804'666	67	-	2	-	Distal Intergenic	LINC02225	GACAGCATAGTGAGCC-ACAAGG	13.5
					OMT12	chr3	133'602'863	133'603'363	9	-	2	-	Intron (intron 1 of 1)	TOPBP1	GGCTGCA--GTGAGCCAAGATCG	10.5
					HMT	chr3	46'356'970	46'358'682	519	-	16	-	5' UTR	CCR2	–	-
					HMT	chr3	52'683'469	52'683'976	488	-	3	-	Promoter (2-3kb)	GNL3	–	-
					HMT	chr5	131'146'598	131'147'098	256	-	3	-	Distal Intergenic	LYRM7	–	-
					HMT	chr5	43'013'395	43'013'897	237	-	3	-	Exon (exon 2 of 2)	ANXA2R-OT1	–	-
					HMT	chr22	23'721'321	23'721'821	273	-	2	-	Distal Intergenic	GUSBP11	–	-

Supplemental Table 3

Component	End Concentration	Company	Catalog number
A83-01 (5 mM)	500 nM	Tocris	2939
Advanced DMEM/F12 +++		Gibco	12634010
B27 (50x)	1x	Thermo Fisher Scientific	17504044
FGF-10	100 ng / mL	PeproTech	100-26
FGF-7	25 ng / mL	PeproTech	100-19
N-Acetylcysteine (500 mM)	1.25 mM	Sigma-Aldrich	A9165-5G
Nicotinamide (1 M)	5 mM	Sigma-Aldrich	N0636-100G
Noggin Conditioned Medium (100%)	5%	Own production	N/A
R-Spondin Conditioned Medium (100%)	5%	Own production	N/A
SB202190 / p38i (30 mM)	500 nM	Sigma-Aldrich	S7067-5MG
Wnt3a Conditioned Medium (100%)	5%	Own production	N/A

Supplemental Table 4

Cell type	Cas9	sgRNA	ssODN	# of nucleofected cells	Format	Kit	Program
K-562	100 pmol	120 pmol	100 pmol	200'000	20 µL Nucleocuvette Strips	SF	FF-120
K-562 FIRE reporter	200 pmol	240 pmol	90 pmol	8'000'000	100 µL single Nucleocuvette	SF	FF-120
K-562 eGFP	100 pmol	120 pmol	100 pmol	200'000	20 µL Nucleocuvette Strips	SF	FF-120
RPE-1 +/- p53	200 pmol	240 pmol	300 pmol	2'000'000	100 µL single Nucleocuvette	P3	EA-104
HSPC	100 pmol	120 pmol	100 pmol	200'000	20 µL Nucleocuvette Strips	P3	ER-100
HSPC (scRNA-seq)	500 pmol	600 pmol	500 pmol	1'000'000	100 µL single Nucleocuvette	P3	ER-100
Upper airway organoid	500 pmol	600 pmol	500 pmol	1'000'000	100 µL single Nucleocuvette	SE	EW-100

Supplemental Table 5

Sanger sequencing		
Target	Primer	Sequence (5' to 3')
FIRE reporter	Forward	GAGAACCATCAGATGTTTCCA
	Reverse	ACGGTGTAGTCCTCGTTGTG

Short-read sequencing		
Target	Primer	Sequence (5' to 3')
HBB	1st PCR - Forward	ACCCTGTTACTTATCCCCTTCC
	1st PCR - Reverse	TCCATCTACATATCCCAAAGCTGA
	2nd nested PCR - Forward	CTTTCCCTACACGACGCTCTTCCGATCTTTCTCTGTCTCCACATGCCC
	2nd nested PCR - Reverse	GGAGTTCAGACGTGTGCTCTTCCGATCTACTGTGTTCACTAGCAACCTCAA
CCR5	Forward	CTTTCCCTACACGACGCTCTTCCGATCTAACTGCAAAAGGCTGAAGAGC
	Reverse	GGAGTTCAGACGTGTGCTCTTCCGATCTCAAACACAGCATGGACGACAG
HBD	Forward	CTTTCCCTACACGACGCTCTTCCGATCTCACACATGACAGAACAGCCAATCTCAG
	Reverse	GGAGTTCAGACGTGTGCTCTTCCGATCTGAAGAAAGTGTAAGCAACAGTCGACTCTG
TRAC	Forward	CTTTCCCTACACGACGCTCTTCCGATCTGCAAAGAGGGAAATGAGATCAT
	Reverse	GGAGTTCAGACGTGTGCTCTTCCGATCTATGTCTAGCACAGTTTTGTCTG
GAPDH	Forward	CTTTCCCTACACGACGCTCTTCCGATCTGGCCTCCAAGGAGTAAGACC
	Reverse	GGAGTTCAGACGTGTGCTCTTCCGATCTCCCAGACCCTAGAATAAGACAGG
AAVS1	Forward	CTTTCCCTACACGACGCTCTTCCGATCTGGGACCACCTTATATTCCCAGG
	Reverse	GGAGTTCAGACGTGTGCTCTTCCGATCTATCCTCTCTGGCTCCATCGT
SMURF1	Forward	CTTTCCCTACACGACGCTCTTCCGATCTCATCCTGTGTCTCATTCACCAT
	Reverse	GGAGTTCAGACGTGTGCTCTTCCGATCTATACCACCACCAGGAAAATCAG
IFT22	Forward	CTTTCCCTACACGACGCTCTTCCGATCTTTCACTCAGACCATCTGTGCTC
	Reverse	GGAGTTCAGACGTGTGCTCTTCCGATCTGAATGAAAATCACATTCAAGCC

Long-read sequencing			
Target	Primer	Amplification size (bp)	Sequence (5' to 3')
FIRE reporter	Forward	3802	TTTCTGTTGGTGCTGATATTGCGAGAACCATCAGATGTTTCCA
	Reverse		ACTTGCCTGTCGCTCTATCTTCGAAGGCGATAGAAGGCGATG
HBB	Forward	4627	TTTCTGTTGGTGCTGATATTGCTCAAGCTACAAAAAGCCGCC
	Reverse		ACTTGCCTGTCGCTCTATCTTCCCTTGAAGCCAGGATGATGGT
CCR5	Forward	4545	TTTCTGTTGGTGCTGATATTGCTTTGGCAAACACCAAGTGCTC
	Reverse		ACTTGCCTGTCGCTCTATCTTCCCCCCTTTGCCTATTGACGG
HBD	Forward	4710	TTTCTGTTGGTGCTGATATTGCTCCTCATGGCCTTAAGAATTTACCT
	Reverse		ACTTGCCTGTCGCTCTATCTTCTGAGGCACAGTAGGCATGTA
TRAC	Forward	5942	TTTCTGTTGGTGCTGATATTGCGGCACATGCAAAGTAGCCTAAG
	Reverse		ACTTGCCTGTCGCTCTATCTTCCGGCCACTTTCAGGAGGAGG
GAPDH	Forward	4690	TTTCTGTTGGTGCTGATATTGCCCCCAGTCTCTGTCCCTTTTG
	Reverse		ACTTGCCTGTCGCTCTATCTTCAGTGCCGTCCGTCTTCAG
AAVS1	Forward	3972	TTTCTGTTGGTGCTGATATTGCCGACCTACTCTCTTCCGCAT
	Reverse		ACTTGCCTGTCGCTCTATCTTCTGCAAACAGGAAGTGAACGG
SMURF1	Forward	4051	TTTCTGTTGGTGCTGATATTGCAGGAACAGACAGACACGCAG
	Reverse		ACTTGCCTGTCGCTCTATCTTCATAACCGGTGCGCAAAGAAA

Digital Droplet PCR		
Target	Primer	Sequence (5' to 3')
U5Psi	Forward	TAGTGTGTGCCCGTCTGTTG
	Reverse	CCTCTGGTTTCCCTTTCGCT
	Probe (5' 6-FAM/ZEN/3' IBFQ)	TCTCTAGCAGTGGCGCCCGA
RPP30	Forward	TGTAAGTGGTAGTGCATAGACTTTA
	Reverse	GTCAAGAGTAGGAGGACATTTGA
	Probe (5' 6-FAM/ZEN/3' IBFQ)	AGGCAGACTGACACTAGAGTTCAC
eGFP	Forward	GCCACAACATCGAGGAC
	Reverse	CTTTACTCAGCTTGGACTGG
	Probe (5' 6-FAM/ 3' BHQ-1)	TCGCCGACCACTACCAGCA
KMT2C	Forward	CGCAGTACCGGAAGATG
	Reverse	GCTTCAGATTGCTAGACTCC
	Probe (5' 6-FAM/ 3' BHQ-1)	AAATCCAATGTGTATCTGGCACGGT
chr3:46,270,956-46,271,086 - used as reference	Forward	AGTAGCATCCAGGCAGGT
	Reverse	ACAGAGAGGCACATTCAAGG
	Probe (5' HEX/ 3' BHQ-1)	TGCCACTGTGCCTTCCAACAAAC

CAST-sequencing			
Target	Function		Sequence (5' to 3')
HBB	CAST-seq PCR I	bait	GTTTCTATTGGTCTCCTTAAACC
		decoy 1	CCATGGTGTCTGTTTGAGG
		decoy 2	CATCTGACTCCTGAGGAGAAG
		decoy ssODN	CACCTGACTCCTGTAGAGAAG
	CAST-seq PCR II	bait nested	GACTGGAGTTCAGACGTGTGCTCTTCCGATCTGGTCTCCTTAAACCTGTCTTGTAACC
CCR5	CAST-seq PCR I	bait	GAAGAAGATTCCAGAGAAGAAGC
		decoy 1	CTCTGACCTGTTTTTCCTTC
		decoy 2	AGGTTGAGCAGGTAGATGTC
	CAST-seq PCR II	bait nested	GACTGGAGTTCAGACGTGTGCTCTTCCGATCTTAGAGCCCTGTCAAGAGTTGACAC
Linker prey primers	CAST-seq PCR I	initial PCR	GTAATACGACTCACTATAGGGC
	CAST-seq PCR II	nested PCR	ACACTCTACACTCTTTCCCTACACGACGCTCTTCCGATCTAGGGCTCCGCTTAAGGGAC
Linker oligo		positive strand	GTAATACGACTCACTATAGGGCTCCGCTTAAGGGACT
		negative strand	P-GTCCCTTAAGCGGAGC-NH3

Supplemental Table 6

Cell type	Target	Condition	inputReads	readsPass Cutadapt1	readsPass Cutadapt2	readsPass Cutadapt3
Figure 1						
RPE-1 p53 -/-	HBB	Unedited	51448	36384	21479	21330
		Cas9	40059	32701	23091	22826
		Cas9 + ssODN	30848	22422	13006	12233
		Cas9 + AZD7648	45519	36806	25405	24743
		Cas9 + ssODN + AZD7648	32522	22654	12601	12272
RPE-1 p53 -/-	CCR5	Unedited	23353	19828	18574	18472
		Cas9	67067	56905	52614	52379
		Cas9 + ssODN	21409	18301	17248	15377
		Cas9 + AZD7648	137854	120057	110917	109484
		Cas9 + ssODN + AZD7648	31405	27155	25496	24870
RPE-1 p53 -/-	GAPDH	Unedited	36680	32493	25177	24984
		Cas9 + ssODN	36264	31627	22117	18288
		Cas9 + ssODN + AZD7648	42306	37588	28255	27464
HSPC Donor #1	HBB	Mock	107130	86953	51442	51369
		Mock + AZD7648	109645	86336	56129	56055
		Cas9 + ssODN	122430	97743	59076	57949
		Cas9 + ssODN + AZD7648	104867	82023	45918	45520
HSPC Donor #1	CCR5	Mock	136455	117142	106409	106085
		Mock + AZD7648	77278	64842	58849	58651
		Cas9 + ssODN	125143	104810	92994	90862
		Cas9 + ssODN + AZD7648	80874	69228	61580	60947
HSPC Donor #1	GAPDH	Mock	96553	70926	50518	50365
		Mock + AZD7648	64766	44731	33487	33373
		Cas9 + ssODN	113171	82474	58746	55640
		Cas9 + ssODN + AZD7648	133949	97246	58650	58099
HSPC Donor #2	HBB	Mock	1096814	452380	339529	338373
		Cas9 + ssODN	753991	304903	244185	239220
		Cas9 + ssODN + AZD7648	834930	345025	275192	272499
HSPC Donor #2	CCR5	Mock	290478	244355	227526	226248
		Cas9 + ssODN	309536	263293	244349	239144
		Cas9 + ssODN + AZD7648	348343	297473	276758	273600
HSPC Donor #2	GAPDH	Mock	834930	343752	282107	280419
		Cas9 + ssODN	792373	334239	271949	258465
		Cas9 + ssODN + AZD7648	757618	307853	246678	244147
Figure 2 and Supplemental Figure 7						
RPE-1 p53 -/-	HBB	Unedited	340866	268001	197215	195644
		Cas9	314815	248458	184497	180268
		Cas9 + PolQi2	339826	259338	185116	182739
		Cas9 + AZD7648	310637	237655	172336	166340
		Cas9 + AZD7648 + PolQi2	301809	210172	123445	122285
		Cas9 + ssODN	159385	120681	89584	82400
		Cas9 + ssODN + PolQi2	165899	121628	83577	79298
		Cas9 + ssODN + AZD7648	328924	247922	173878	164849
		Cas9 + ssODN + AZD7648 + Pol(	218457	167092	112803	111392
RPE-1 p53 -/-	CCR5	Unedited	342684	286704	271040	269855
		Cas9	174978	145221	137362	135817
		Cas9 + PolQi2	199338	167401	158270	157094
		Cas9 + AZD7648	251690	215655	203992	200481
		Cas9 + AZD7648 + PolQi2	253656	217968	206103	205087
		Cas9 + ssODN	296829	248854	235098	215462
		Cas9 + ssODN + PolQi2	509624	435905	412306	381743
		Cas9 + ssODN + AZD7648	198386	168252	158608	153064
		Cas9 + ssODN + AZD7648 + Pol(	260642	215564	203894	201854
RPE-1 p53 -/-	GAPDH	Unedited	116065	98080	85608	68970
		Cas9	121258	102898	90351	77032
		Cas9 + PolQi2	129139	107848	93413	79752
		Cas9 + AZD7648	157395	135695	117325	114839
		Cas9 + AZD7648 + PolQi2	187184	164246	142601	140572
		Cas9 + ssODN	149607	127733	110604	81617
		Cas9 + ssODN + PolQi2	135976	111437	96977	69689
		Cas9 + ssODN + AZD7648	192984	170424	146961	132534
		Cas9 + ssODN + AZD7648 + Pol(	104285	78335	67915	55814
RPE-1 p53 +/+	HBB	Unedited	424351	318215	213162	211290
		Cas9	509553	391824	282866	278235
		Cas9 + PolQi2	262685	191808	127289	125821
		Cas9 + AZD7648	354675	256980	165911	161885
		Cas9 + AZD7648 + PolQi2	383608	243404	122085	120822
		Cas9 + ssODN	500698	369989	257152	235274
		Cas9 + ssODN + PolQi2	224238	152245	100262	93514
		Cas9 + ssODN + AZD7648	743518	504721	280215	268141
		Cas9 + ssODN + AZD7648 + Pol(	227724	158232	88194	86493
RPE-1 p53 +/+	CCR5	Unedited	308372	258391	243790	242754
		Cas9	417016	358507	339378	336668
		Cas9 + PolQi2	352687	298278	283366	281876
		Cas9 + AZD7648	341542	291152	276364	272653
		Cas9 + AZD7648 + PolQi2	376608	324801	308353	307004
		Cas9 + ssODN	323025	269569	255380	234559
		Cas9 + ssODN + PolQi2	200295	165362	156702	145200
		Cas9 + ssODN + AZD7648	372164	318863	302075	294286
		Cas9 + ssODN + AZD7648 + Pol(	376756	325198	308954	305919
RPE-1 p53 +/+	GAPDH	Unedited	116065	98080	85608	68970
		Cas9	111580	95241	82926	68940
		Cas9 + PolQi2	113727	95551	83162	69407
		Cas9 + AZD7648	127254	111378	97959	91329
		Cas9 + AZD7648 + PolQi2	161277	140679	123055	106820
		Cas9 + ssODN	81895	69885	61661	45476
		Cas9 + ssODN + PolQi2	131711	113714	99788	74569
		Cas9 + ssODN + AZD7648	209864	178478	155909	124748
		Cas9 + ssODN + AZD7648 + Pol(	123136	101612	89837	71184

Cell type	Target	Condition	inputReads	readsPass Cutadapt1	readsPass Cutadapt2	readsPass Cutadapt3
Supplemental Figure 3						
K-562 FIRE reporter	FIRE reporter	Unedited	66843	57069	45326	45292
		Cas9	82766	68164	53254	53031
		Cas9 + ssODN	64571	52260	41244	39904
		Cas9 + AZD7648	35532	30613	23083	23043
		Cas9 + ssODN + AZD7648	114446	91807	71064	64557
RPE-1 p53 -/-	TRAC	Unedited	68052	53961	47368	47131
		Cas9	60885	49743	43282	42772
		Cas9 + ssODN	57603	46804	40333	38877
		Cas9 + AZD7648	43409	34258	29546	29341
		Cas9 + ssODN + AZD7648	57801	46855	40474	39921
RPE-1 p53 -/-	HBD	Unedited	26275	22908	20116	20055
		Cas9	43028	34632	30502	30355
		Cas9 + ssODN	17568	15233	13569	12388
		Cas9 + AZD7648	38904	32341	28518	27938
		Cas9 + ssODN + AZD7648	21214	18610	16570	15287
RPE-1 p53 -/-	AAVS1	Unedited	161323	138279	125341	124767
		Cas9	100047	84219	73136	72851
		Cas9 + ssODN	97640	77980	66538	66039
		Cas9 + AZD7648	171397	140654	113993	113665
		Cas9 + ssODN + AZD7648	125999	105121	90196	89899
RPE-1 p53 +/+	TRAC	Unedited	68810	55834	49042	48785
		Cas9	42565	33734	29166	28958
		Cas9 + ssODN	103386	55110	34935	34368
		Cas9 + AZD7648	98580	42871	27069	26937
		Cas9 + ssODN + AZD7648	140568	59506	24126	23871
RPE-1 p53 +/+	HBD	Unedited	23812	20647	18768	18704
		Cas9	75056	62784	54774	54479
		Cas9 + ssODN	20393	17771	15557	13500
		Cas9 + AZD7648	75986	64715	57173	56214
		Cas9 + ssODN + AZD7648	29981	26271	23386	22321
RPE-1 p53 +/+	AAVS1	Unedited	105879	89664	80389	80019
		Cas9	103377	86794	77122	76940
		Cas9 + ssODN	62674	50276	43638	43279
		Cas9 + AZD7648	119915	102005	90846	90573
		Cas9 + ssODN + AZD7648	413319	168496	31512	31222
RPE-1 p53 +/+	CCR5	Unedited	21820	18440	17401	17316
		Cas9	42694	35672	33040	32918
		Cas9 + ssODN	23975	20308	19053	17139
		Cas9 + AZD7648	56005	47685	43963	43539
		Cas9 + ssODN + AZD7648	13633	11466	10797	10638
RPE-1 p53 +/+	GAPDH	Unedited	16393	13849	7383	7338
		Cas9 + ssODN	37675	33155	24929	21586
		Cas9 + ssODN + AZD7648	24531	21555	11651	11330
RPE-1 p53 +/+	HBB	Unedited	20999	15815	9798	9742
		Cas9	32130	19654	8385	8279
		Cas9 + ssODN	76478	48307	27400	25959
		Cas9 + AZD7648	32034	19839	10189	10087
		Cas9 + ssODN + AZD7648	62967	38735	19718	19306
K-562	TRAC	Unedited	89588	71210	62236	62061
		Cas9	68840	55795	48212	47919
		Cas9 + ssODN	63456	49706	42274	42024
		Cas9 + AZD7648	70081	57883	49032	48798
		Cas9 + ssODN + AZD7648	75074	60530	52383	52248
K-562	HBD	Unedited	25965	20977	19148	19080
		Cas9	11933	9455	8581	8498
		Cas9 + ssODN	28471	23314	21159	20193
		Cas9 + AZD7648	20699	17104	15575	15446
		Cas9 + ssODN + AZD7648	17555	14252	12965	12244
K-562	AAVS1	Unedited	209972	178609	158824	158085
		Cas9	133925	112911	98519	97778
		Cas9 + ssODN	226210	187480	157990	156459
		Cas9 + AZD7648	299531	255529	221310	220386
		Cas9 + ssODN + AZD7648	210163	178485	154425	153026
K-562	CCR5	Unedited	36936	31264	29471	29360
		Cas9	21863	18477	17291	17180
		Cas9 + ssODN	21959	18642	17585	17083
		Cas9 + AZD7648	30152	25917	24327	24165
		Cas9 + ssODN + AZD7648	24630	20873	19594	19286
K-562	GAPDH	Cas9	241722	181173	159998	159024
		Cas9 + ssODN	145456	111209	99030	96229
		Cas9 + AZD7648	82207	60928	51789	51482
		Cas9 + ssODN + AZD7648	132470	112029	103640	102838
Supplemental Figure 8						
K-562 eGFP	SMURF1	Unedited	492713	389666	357719	356306
		Cas9	480658	394176	362323	358711
		Cas9 + PolQi2	428690	333268	300036	297324
		Cas9 + AZD7648	327643	250865	218636	216392
		Cas9 + AZD7648 + PolQi2	440311	224618	140036	138789
		Cas9 + ssODN	196277	142150	121060	116910
		Cas9 + ssODN + PolQi2	213659	156587	137737	133407
		Cas9 + ssODN + AZD7648	498222	387963	347064	342931
		Cas9 + ssODN + AZD7648 + Pol(	242463	180790	160195	158458

Supplemental Table 7

Target	cutadapt1_ForwardStrand		cutadapt1_ReverseStrand		cutadapt2_ForwardStrand		cutadapt2_ReverseStrand		PCR products size (bp)		
	stubForward	stubReverse	stubForward	stubReverse	gDNA5	gDNA3	gDNA5	gDNA3	w/o stub	w/ stub	w/o stub & gDNA primer
HBB	TTTCTGTTGGTGCTGATATTGCTCAAGCTACAAAAAGCCGCC	ACCATCATCCTGGCTTCAAGGGAAGATAGAGCGACAGGCAAGT	ACTTGCCTGTCGCTCTATCTTCCCTTGAAGCCAGGATGATGGT	GGCGGCTTTTTGTAGCTTGAGCAATATCAGCACCAACAGAAA	TTTCAAATTCTTCTCAGTCCTAACTTTTCA	CCTTCTCTTGTGTTGGCAACTGCTGCAGAT	ATCTGCAGCAGTTGCCAACACAAGAGAAGG	TGAAAAGTTAGGACTGAGAAGAATTTGAAA	4583	4627	4542
HBD	TTTCTGTTGGTGCTGATATTGCTCCTCATGGCCTTAAGAATTTACCT	TACATGCCTACTGTGCCTCAGAAGATAGAGCGACAGGCAAGT	ACTTGCCTGTCGCTCTATCTTCTGAGGCACAGTAGGCATGTA	AGGTAAATTCTTAAGGCCATGAGGAGCAATATCAGCACCAACAGAAA	AACATTTTTTTTAGAATCAATTTTCTTATA	ATAAGCAATTCTGCCTATTCTCTACCTTCT	AGAAGGTAGAGAATAGGCAGAATTGCTTAT	TATAAGAAAATTGATTCTAAAAAAAATGTT	4666	4710	4621
CCR5	TTTCTGTTGGTGCTGATATTGCTTTGGCAAACACCAAGTGCTC	CCGTCAATAGGCAAAGGGGGGAAGATAGAGCGACAGGCAAGT	ACTTGCCTGTCGCTCTATCTTCCCCCCTTTGCCTATTGACGG	GAGCACTTGGTGTTTGCCAAAGCAATATCAGCACCAACAGAAA	ATACAATTATCTTAAAATATAATCTTTAAG	ACAAGTGTAGGTATCATTTTCTGCATTTAA	TTAAATGCAGAAAATGATACCTACACTTGT	CTTAAAGATTATATTTTAAGATAATTGTAT	4501	4545	4460
GAPDH	TTTCTGTTGGTGCTGATATTGCCCCCAGTCTCTGTCCCTTTTG	CTGAAGACGGACGGCACTGAAGATAGAGCGACAGGCAAGT	ACTTGCCTGTCGCTCTATCTTCAGTGCCGTCCGTCTTCAG	CAAAAGGGACAGAGACTGGGGGCAATATCAGCACCAACAGAAA	TAGGAGGGACTTAGAGAAGGGGTGGGCTTG	CAGTTGTCAACCTTGGGGCAGGCAGGAATC	GATTCCTGCCTGCCCCAAGGTTGACAACTG	CAAGCCCACCCCTTCTCTAAGTCCCTCCTA	4646	4690	4586
TRAC	TTTCTGTTGGTGCTGATATTGCGGCACATGCAAAGTAGCCTAAG	CCTCCTCCTGAAAGTGGCCGGAAGATAGAGCGACAGGCAAGT	ACTTGCCTGTCGCTCTATCTTCCGGCCACTTTCAGGAGGAGG	CTTAGGCTACTTTGCATGTGCCGCAATATCAGCACCAACAGAAA	TATACATCTGATAATAAAATTGGTTGAAAA	TCAAAACCTGTCAGTGATTGGGTTCCGAAT	ATTCGGAACCCAATCACTGACAGGTTTTGA	TTTTCAACCAATTTTATTATCAGATGTATA	5898	5942	5856
AAVS1	TTTCTGTTGGTGCTGATATTGCCGACCTACTCTCTTCCGCAT	CCGTTCACTTCCTGTTTGCAGAAGATAGAGCGACAGGCAAGT	ACTTGCCTGTCGCTCTATCTTCTGCAAACAGGAAGTGAACGG	ATGCGGAAGAGAGTAGGTCGGCAATATCAGCACCAACAGAAA	TGGAGTCGCTTTAACTGGCCCTGGCTTTGG	CGCACCCAGATGAGAAGCCCCCTCCCTTCC	GGAAGGGAGGGGGCTTCTCATCTGGGTGCG	CCAAAGCCAGGGCCAGTTAAAGCGACTCCA	3928	3972	3888
FIRE reporter	TTTCTGTTGGTGCTGATATTGCGAGAACCATCAGATGTTTCCA	CATCGCCTTCTATCGCCTTCGAAGATAGAGCGACAGGCAAGT	ACTTGCCTGTCGCTCTATCTTCGAAGGCGATAGAAGGCGATG	TGGAAACATCTGATGGTTCTCGCAATATCAGCACCAACAGAAA	GGGTGCCCCAAGGACCTGAAATGACCCTGT	TTACGGTATCGCCGCTCCCGATTCGCAGCG	CGCTGCGAATCGGGAGCGGCGATACCGTAA	ACAGGGTCATTTCAGGTCCTTGGGGCACCC	3758	3802	3717
SMURF1	TTTCTGTTGGTGCTGATATTGCAGGAACAGACAGACAC[illegible]	TTTCTTTGCGCACCGGTTATGAAGATAGAGCGACAGGC[illegible]	ACTTGCCTGTCGCTCTATCTTCATAACCGGTGCGCAAA[illegible]	CTGCGTGTCTGTCTGTTCCTGCAATATCAGCACCAACA[illegible]	TACATCGTTAGTGGTGACGTTGCCTGACAG	GAAAAAACATGGGATTGAGAAAATTTAGAA	TTCTAAATTTTCTCAATCCCATGTTTTTTC	CTGTCAGGCAACGTCACCACTAACGATGTA	4007	4051	3967
