# Supplementary material for: Genome editing with the HDR-enhancing DNA-PKcs inhibitor AZD7648 causes large-scale genomic alterations
Source: Nat Biotechnol. 2024 Nov 27;43(11):1778–82. doi: 10.1038/s41587-024-02488-6 (PMC12611759; doi:10.1038/s41587-024-02488-6)
Supplement: Supplementary file 2 — Reporting Summary [file 41587_2024_2488_MOESM2_ESM.pdf]

nature portfolio

Corresponding author(s): Jacob E. Corn

Last updated by author(s): Oct 17, 2024

Reporting Summary

Nature Portfolio wishes to improve the reproducibility of the work that we publish. This form provides structure for consistency and transparency in reporting. For further information on Nature Portfolio policies, see our Editorial Policies and the Editorial Policy Checklist.

Statistics

For all statistical analyses, confirm that the following items are present in the figure legend, table legend, main text, or Methods section.

n/a	Confirmed	
☐	☒	The exact sample size (*n*) for each experimental group/condition, given as a discrete number and unit of measurement
☐	☒	A statement on whether measurements were taken from distinct samples or whether the same sample was measured repeatedly
☒	☐	The statistical test(s) used AND whether they are one- or two-sided<br>*Only common tests should be described solely by name; describe more complex techniques in the Methods section.*
☒	☐	A description of all covariates tested
☒	☐	A description of any assumptions or corrections, such as tests of normality and adjustment for multiple comparisons
☐	☒	A full description of the statistical parameters including central tendency (e.g. means) or other basic estimates (e.g. regression coefficient) AND variation (e.g. standard deviation) or associated estimates of uncertainty (e.g. confidence intervals)
☐	☒	For null hypothesis testing, the test statistic (e.g. *F*, *t*, *r*) with confidence intervals, effect sizes, degrees of freedom and *P* value noted<br>*Give P values as exact values whenever suitable.*
☐	☒	For Bayesian analysis, information on the choice of priors and Markov chain Monte Carlo settings
☒	☐	For hierarchical and complex designs, identification of the appropriate level for tests and full reporting of outcomes
☒	☐	Estimates of effect sizes (e.g. Cohen's *d*, Pearson's *r*), indicating how they were calculated

Our web collection on statistics for biologists contains articles on many of the points above.

Software and code

Policy information about availability of computer code

Data collection	ddPCR data was collected using QX200 Droplet Reader and QuantaSoft software v1.7.4.0917 (BioRad). Chromium Controller (Firmware v4.0) was used for scRNA-seq. Genomic DNA was quantified using using NanoDrop 8000 Spectrophotometer or Qubit Fluorometers (APP 2.02 + MCU v0.26). PCR products were quantified using Qubit 4.0 Fluorometer (APP 2.02 + MCU v0.26) or Victor NIVO multimode plate reader with Control software v4.0.7 and characterized using Agilent Tapestation software v5.1. Flow cytometry data were collected using the Attune NxT Cytometer (with autosampler) and Attune NxT Software v3.2.1. For cell sorting experiments, the Sony SH800S device and Cell Sorter Software v2.1.6 were used.
Data analysis	Microsoft® Excel for Mac (v16.84) and GrapPad Prism v10.0.3 (217) for Mac were used for data analysis and figures. Flow cytometry data were analyzed using FlowJo v10.8.2. Short-read sequencing were analyzed using CRISPResso2 (v2.0.20b) and ICE Synthego v3.0 for indel analysis. Long-read sequencing were analyzed using cutadapt (version 4.6), minimap 2 (version 2.24-r1122), deepTools (version 3.5.4), bioawk (version 1.0) and described at https://github.com/cornlab/summarizeOntDeletions. R (4.2.1), infercnv(1.12.0), Seurat (5.1.0), dplyr (1.1.4), ggplot2 (3.5.1), ComplexHeatmap (2.20.0), were used for single-cell RNA sequencing analysis. CAST-seq were analyzed as described at https://github.com/AG-Boerries/CAST-Seq

For manuscripts utilizing custom algorithms or software that are central to the research but not yet described in published literature, software must be made available to editors and reviewers. We strongly encourage code deposition in a community repository (e.g. GitHub). See the Nature Portfolio guidelines for submitting code & software for further information.

Data

Policy information about availability of data

All manuscripts must include a data availability statement. This statement should provide the following information, where applicable:

- Accession codes, unique identifiers, or web links for publicly available datasets
- A description of any restrictions on data availability
- For clinical datasets or third party data, please ensure that the statement adheres to our policy

Sequencing data for short-read, long-read, and CAST-sequencing are available in the Sequence Read Archive under BioProject PRJNA1167903. GRCh38 was downloaded from GenBank. All the raw data supporting the findings of this study are available from the corresponding author upon reasonable request.

Research involving human participants, their data, or biological material

Policy information about studies with human participants or human data. See also policy information about sex, gender (identity/presentation), and sexual orientation and race, ethnicity and racism.

Reporting on sex and gender	The primary cell samples used in this study were de-identified, so we do not have information on the donors' gender.
Reporting on race, ethnicity, or other socially relevant groupings	The primary cell samples used in this study were de-identified, so we have no information on the race, ethnicity or other socially relevant groupings.
Population characteristics	The primary cell samples used in this study were de-identified, so we have no additional information on the population characteristics, other than the fact that they came from adult donors.
Recruitment	The human G-CSF-mobilized CD34+ HSPC from adult healthy donors were purchased from the Fred Hutchinson Cancer Center. The human nasal epithelial samples were collected from healthy volunteers.
Ethics oversight	The human nasal epithelial samples were collected in accordance with ethical guidelines of ETH Zürich Ethics Commission (EK-2024-N-171-A) and Cantonal Ethics Committee Zürich (Req-2O24-OO558).

Note that full information on the approval of the study protocol must also be provided in the manuscript.

Field-specific reporting

Please select the one below that is the best fit for your research. If you are not sure, read the appropriate sections before making your selection.

☒ Life sciences ☐ Behavioural & social sciences ☐ Ecological, evolutionary & environmental sciences

For a reference copy of the document with all sections, see nature.com/documents/nr-reporting-summary-flat.pdf

Life sciences study design

All studies must disclose on these points even when the disclosure is negative.

Sample size	No formal sample size calculations were performed. The sample size was considered sufficient based on consistent measurable differences observed and in line with previously published studies conducting similar analyses.
Data exclusions	No data were excluded from the analyses
Replication	Replicates were not performed, except for the gene editing assay using flow cytometry as the readout. In this case, the number of individual biological replicates is specified in the figure legends.
Randomization	No randomization was performed. All independent biological experiments were treated equally
Blinding	Blinding was not performed as the data were not subjective.

Reporting for specific materials, systems and methods

We require information from authors about some types of materials, experimental systems and methods used in many studies. Here, indicate whether each material, system or method listed is relevant to your study. If you are not sure if a list item applies to your research, read the appropriate section before selecting a response.

Materials & experimental systems

n/a	Involved in the study	
☒	☐	Antibodies
☐	☒	Eukaryotic cell lines
☒	☐	Palaeontology and archaeology
☒	☐	Animals and other organisms
☒	☐	Clinical data
☒	☐	Dual use research of concern
☒	☐	Plants

Methods

n/a	Involved in the study	
☒	☐	ChIP-seq
☐	☒	Flow cytometry
☒	☐	MRI-based neuroimaging

Eukaryotic cell lines

Policy information about cell lines and Sex and Gender in Research

Cell line source(s)	The hTERT RPE-1 p53+/+ and hTERT RPE-1 p53-/- cells were a gift from Stephen Jackson (with hTERT RPE-1 originally purchased from ATCC).<br>The human G-CSF-mobilized CD34+ HSPC from adult healthy donors were purchased from the Fred Hutchinson Cancer Center.<br>The human nasal epithelial samples were collected from healthy volunteers.<br>The K-562 cells were purchased from ATCC
Authentication	The hTERT RPE-1 p53+/+, hTERT RPE-1 p53-/-, K-562, and K-562 eGFP cell lines were STR-profiled.
Mycoplasma contamination	The hTERT RPE-1 p53+/+, hTERT RPE-1 p53-/-, K-562, and K-562 eGFP cell lines were routinely tested negative for mycoplasma (MycoAlert, Lonza).
Commonly misidentified lines<br>(See ICLAC register)	No commonly misidentified cell lines were used.

Plants

Seed stocks	N/A
Novel plant genotypes	N/A
Authentication	N/A

Flow Cytometry

Plots

Confirm that:

☒ The axis labels state the marker and fluorochrome used (e.g. CD4-FITC).

☒ The axis scales are clearly visible. Include numbers along axes only for bottom left plot of group (a 'group' is an analysis of identical markers).

☒ All plots are contour plots with outliers or pseudocolor plots.

☒ A numerical value for number of cells or percentage (with statistics) is provided.

Methodology

Sample preparation	The FIRE K-562 and eGFP K-562 were directly transferred for analysis. Cell viability of GAPDH-edited HSPCs analyzed by single-cell RNA sequencing was also quantified using SYTOX™ Red Dead Cell Stain (1:1000 dilution).
Instrument	Attune NxT Cytometer (with autosampler) and SH800S Cell Sorter (Sony) for cell sorting.
Software	Attune NxT Software v3.2.1. and Cell Sorter Software v2.1.6
Cell population abundance	Purity of sorted fractions was not quantified.

Gating strategy

Cells were first gated on morphology (FSC-A vs SSC-A), for single cells (FSC-A vs FSC-H) and then on mScarlet and eGFP expression. The gating strategy is shown in Extended Data Figure 2b.
Edited K-562 cells expressing eGFP were first gated on morphology (FSC-A vs SSC-A), for single cells (FSC-A vs FSC-H) and then on eGFP expression. The gating strategy is shown in Extended Data Figure 4c.
Edited HSPCs were first gated on morphology (FSC-A vs SSC-A), for single cells (FSC-A vs FSC-H) and then on SYTOX™ Red Dead Cell Stain level. The gating strategy is shown in Extended Data Figure 5a.

☒ Tick this box to confirm that a figure exemplifying the gating strategy is provided in the Supplementary Information.
